# Gut Molecules in Cardiometabolic Diseases: The Mechanisms behind the Story

**DOI:** 10.3390/ijms24043385

**Published:** 2023-02-08

**Authors:** Andreea-Ioana Inceu, Maria-Adriana Neag, Anca-Elena Craciun, Anca-Dana Buzoianu

**Affiliations:** 1 Department of Pharmacology, Toxicology and Clinical Pharmacology, Iuliu Hatieganu University of Medicine and Pharmacy, 400337 Cluj-Napoca, Romania; 2 Department of Diabetes, and Nutrition Diseases, Iuliu Hatieganu University of Medicine and Pharmacy, 400006 Cluj-Napoca, Romania

**Keywords:** gut hormones, gut microbiota, atherosclerosis, heart failure, atrial fibrillation, diabetes mellitus, inflammation

## Abstract

Atherosclerotic cardiovascular disease is the most common cause of morbidity and mortality worldwide. Diabetes mellitus increases cardiovascular risk. Heart failure and atrial fibrillation are associated comorbidities that share the main cardiovascular risk factors. The use of incretin-based therapies promoted the idea that activation of alternative signaling pathways is effective in reducing the risk of atherosclerosis and heart failure. Gut-derived molecules, gut hormones, and gut microbiota metabolites showed both positive and detrimental effects in cardiometabolic disorders. Although inflammation plays a key role in cardiometabolic disorders, additional intracellular signaling pathways are involved and could explain the observed effects. Revealing the involved molecular mechanisms could provide novel therapeutic strategies and a better understanding of the relationship between the gut, metabolic syndrome, and cardiovascular diseases.

## 1. Introduction

Atherosclerosis and its main consequences, coronary and cerebrovascular artery disease, are a common cause of morbidity and mortality worldwide. Ischemic heart disease is the most common cause of death, responsible for 20% of deaths in Europe, and its frequency is increasing. Cerebrovascular disease, causing 11% of all deaths, carries the risk of high morbidity and decreased quality of life [1]. Atherosclerotic lesion histology progresses over time while complex pathways are activated. The evolution of atherosclerotic plaques may be surprisingly accelerated behind the guidelines-recommended treatment of cardiovascular risk factors, revealing that untargeted processes are activated [2].

Heart failure (HF) is a clinical syndrome, affecting 1–2% of adults with an incidence that is increasing overall [3]. Traditionally, it can be classified based on the left ventricular ejection fraction (LVEF), with treatment strategies recommended by the guidelines for each category [4]. Although the current therapeutical classes target well-known pathophysiological pathways, mortality during the long-term follow-up in the next 5 years remains high, affecting more than 60% in some populations [5], suggesting the need for a more broadened perspective on the pathological processes involved. 

Diabetes mellitus is defined and diagnosed as a presence of high serum concentrations of glucose due to dysregulated insulin secretion and/or insulin sensitivity and can be classified according to different pathophysiology processes, with different therapeutic management solutions [6]. However, impaired glucose metabolism increases the risk of cardiovascular diseases. The use of currently recommended drugs in the treatment of diabetes mellitus evidenced surprisingly beneficial cardiovascular actions [7]. Further investigations regarding the multimolecular mechanisms are required to better understand the long-term effects of antidiabetic drugs.

Gut hormones are peptides secreted from the specialized enteroendocrine cells (EECs) distributed among epithelial cells within the mucosa of the small and large intestines. Following nutrient ingestion, the interaction between the gut cells, the biochemical components of the nutrients, and the local secretions activate EECs to release gastrointestinal hormones, with the role to mediate appetite, intestinal functions, and postprandial metabolism. EECs found in distinctive regions of the gut produce different hormones according to the intraluminal content, suggesting that the secretion profile of gut peptides provides strictly controlled metabolic effects. Besides their metabolic effects, gut peptides mediate systemic functions based on the bonding of specialized receptors. These receptors activate downstream signaling pathways in the peripheric tissues that are involved in the development of cardiometabolic diseases [8]. 

Gut microbiota comprises all the microorganisms that reside in the intestine. In healthy conditions, the gut microbiota assures a normal gastrointestinal function along a cohesive epithelial barrier and controlled local immunity. Gut microorganisms regulate many gastrointestinal and systemic functions through local mechanisms and the release of intestinal microbiota-derived metabolites. These metabolites can act both in a paracrine and endocrine way and define the complex relationship between gut microbiota and cardiometabolic diseases. Gut dysbiosis often referred to as changes in gut microbiota composition may lead to impaired function of the gut barrier, known as leaky gut. In the leaky gut state, the translocation of bacterial products into the systemic circulation is increased and drives to a pro-inflammatory state related to increased cardiovascular risk [7]. However, the precise molecular mechanisms of this association are not fully elucidated. 

Therefore, a better look at the cardiometabolic properties of gut metabolites, including gut peptides and microbiota-derived products, is needed to develop novel therapies for an integrated cardiovascular risk approach. This review will focus on the main molecular mechanisms that drive the cardiac, vascular, inflammatory, and pancreatic properties of gut molecules.

## 2. Gut Peptides: The Mechanisms behind the Molecules 

Gut hormones are peptides secreted by the EECs in response to the presence of small particles derived from the ingested food in the gut. Gut peptides regulate digestive functions, control glycemic metabolism, and maintain energy metabolism through bonding to their specific receptors [8]. A summary of the gut molecules, the bonding receptors, and consecutive metabolic pancreatic functions is provided in Table 1.

- Incretins

Incretins, named after the incretin effect, are a group of intestinal peptides, such as glucagon-like peptide-1 (GLP-1) and gastric inhibitory peptide (GIP), involved in postprandial glucose metabolism. Incretins are secreted as a response to nutrient ingestion, mainly glucose, and are involved in the increase in glucose-induced insulin secretion [18]. The incretin effect is defined as the difference in insulin secretion after oral glucose ingestion and iso-glycemic intravenous glucose infusion, a difference based on intestinal cells’ ability to secrete different peptides after nutrient ingestion. In a review of several studies using incretins receptor antagonists, such as GIP receptor antagonist GIP(3–30)NH2 and GLP-1 receptor antagonist exendin(9–39)NH2, it was demonstrated that in healthy individuals, GIP and GLP-1 are responsible for 60–75% of the insulin secretion following meal ingestion. GIP seemed to be the most important regarding the incretin effect of postprandial glycemia control [19]. The first identified incretin was gastric inhibitory peptide (GIP) in the 1970s by Brown et al. It is a 43-amino acid straight-chain polypeptide isolated from the porcine small intestine and it was first observed to have an inhibitory effect on the secretion of gastric acid [20]. GIP is a member of the glucagon superfamily and it was later discovered to be secreted from the K cells in the proximal part of the small intestine and be involved in the modulation of insulin secretion [21]. In a study that enrolled control subjects and patients with different gastrectomy procedures, Takemura et al. observed that endogenous GIP is a glucose-dependent insulinotropic factor, so GIP was renamed as a glucose-dependent insulinotropic polypeptide to retain the existing acronym [22]. 

Glucagon-like peptide-1 (GLP-1) is the second incretin identified in 1983 [23]. GLP-1 is a 30 (7–36 amide)- or 31 (7–37 amide)-amino acid residue peptide hormone released mainly from intestinal L-cells in the distal intestine after nutrient consumption [24]. It can also be secreted by other tissues, such as pancreatic alpha-cells, and the central nervous system. It is an important hormone linking nutrient consumption with glucose control by promoting the glucose-induced secretion of insulin from pancreatic beta-cells, inhibiting beta-cell apoptosis, promoting beta-cell neogenesis, increasing insulin expression, and reducing glucagon secretion [25]. It also affects the motility of the digestive tract by delaying gastric emptying and on the central nervous system by promoting satiety, with a valuable effect on reducing food intake and consecutive weight loss [26].

### 2.1. GLP-1

GLP-1 is released from intestinal L cells, endocrine cells of the gut epithelium, as a product of the proglucagon gene. Prohormone convertase PC 1/3 produces a proteolytic cleavage of the proglucagon precursor and results in GLP-1(7–37) and GLP-1-(7–36)NH. GLP-1 levels increase rapidly by approximately 10 min after meal ingestion. GLP-1 secretion relies on the entry of glucose in L cells, secondary to nutrient absorption regarding carbohydrates and proteins and direct sensing of L cell receptors located in the intestinal lumen for some lipids. Moreover, GLP-1 is involved in the ileal break, meaning that the presence of nutrients in the distal segments of the small intestine increases the secretion of GLP-1, resulting in the decrease in upper gastrointestinal digestive activities, such as motility and secretion, and inhibition of appetite [18]. 

After release, GLP-1 diffuses across the lamina propria and enters the capillary mucosa, where it is degraded extensively by dipeptidyl peptidase-4 (DPP-4) found in endothelial cells. Furthermore, in the liver, DPP-4 degrades approximately half of the remaining GLP-1, and plasmatic DPP-4 causes more degradation, resulting in approximately 8% of the original release reaching the target organs in the intact form, as determined in experimental studies [27]. The receptors of β cells are very sensitive to GLP-1 and the small amount of GLP-1 that reaches pancreatic tissue can provide endocrine effects [18]. Since the discovery of extensive and rapid 1–2 min degradation of GLP-1, there have been assumptions regarding the alternative signaling pathways, particularly via enteric nervous system components; the sensory afferents fibers that connected with the nucleus of the solitary tract and vagus nerve efferent fibers that reach the peripheral organs [18]. GLP-1 bioavailability is also controlled by some immune cells from the small intestine enterocyte layer, particularly integrin β7+ natural gut intraepithelial T lymphocytes (natural intraepithelial lymphocytes -IEL) via IEL GLP-1 receptor expression [28]. 

GLP-1 exerts several biological effects through binding to its receptor, GLP-1 receptor (GLP-1R). The structure of GLP-1R is similar to the structure of glucagon receptor (GCGR), both being part of the class B family of G-protein coupled receptors (GPCR). Using computational methods, including homology modeling, protein–protein docking, and molecular dynamics simulation, it was indicated that the absence or presence of GLP-1 can change the conformation of the GLP-1R in a closed or open state, respectively, affecting the extracellular domain. GLP-1 exhibits a dynamic molecular nature, with two major forms, the straight or bent helix, and depending on the structure, it induces the open state of the receptor [29].

The activation of GLP-1R in pancreatic tissue enhances insulin biosynthesis and secretion by activating adenylate cyclase and further production of cyclic AMP (cAMP). cAMP stimulates insulin secretion through protein kinase A (PKA)- dependent phosphorylation and exchange protein directly activated by cAMP 2 (EPAC2) activation. These effectors promote insulin secretion via inhibition of K-ATP channels and stimulation of mitochondrial ATP synthesis with subsequent β-cells depolarization, an increase in intracellular Ca^2+^ that leads to storage granule exocytosis and delay in cell repolarization with a decrease in voltage-dependent K (Kv) currents [9]. Insulin biosynthesis is stimulated via cAMP/PKA-dependent mechanisms and EPAC2 activation, leading to an increase in the level of intracellular calcium and nuclear factor of activated T (NFAT) activation. Pancreas duodenum homeobox 1 (Pdx-1), a transcription factor involved in pancreatic cell function, can be activated by GLP-1 signaling, and promotes insulin biosynthesis, secretion, β-cell survival, proliferation, and differentiation [10]. Furthermore, GLP-1 improves the glucose sensibility of glucose-resistant-β-cell via up-regulation of glucose transport and glucokinases and K-ATP channel regulation [11]. 

GLP-1R activation mediates β-cells cytoprotection and proliferation, improving insulin-dependent metabolism. GLP-1 favors β-cell proliferation and neogenesis via Phosphatidylinositol-4,5-bisphosphate 3-kinase (PI-3K) and cAMP/PKA/mitogen-activated protein kinase (MAPK)/cyclin D1 pathways. The PI-3K-mediated pathway involves the activation of the epidermal growth factor receptor (EGFR) and further stimulation of protein kinase C (PKC) ζ and/or Akt-protein kinase B (Akt-PKB) [11]. 

**Figure 1:**
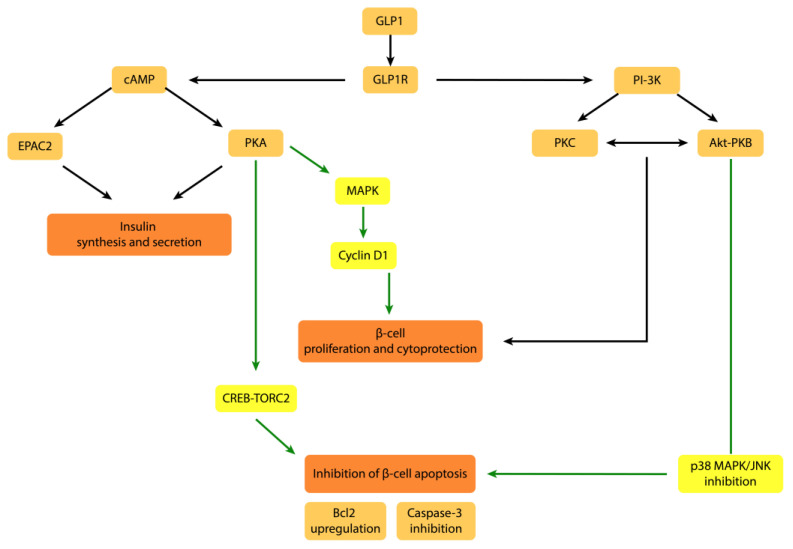
The signaling pathways involved in GLP-1 protective effects in the pancreatic β-cells. The activation of GLP-1R leads to multiple effects in β-2 cells. The direct signaling pathways are represented in black arrows and the mediators involved are depicted in orange boxes. The indirect molecular cascades comprise green arrows that involve additional mediators represented in yellow boxes. The two-headed arrow represents the bidirectional relationship between PKC and Akt-PKB. GLP-1: glucagon-like peptide-; GLP-1R: GLP-1 receptor; cAMP: Adenosine 3’,5’-cyclic monophosphate; PKA: protein kinase A; EPAC2: exchange protein directly activated by cAMP 2; MAPK: mitogen-activated protein kinase; CREB: cAMP response element binding protein; TORC2: transducer of regulated CREB activity 2; PI-3K: Phosphatidylinositol-4,5-bisphosphate 3-kinase; PKC: protein kinase C; Akt-PKB: Akt-protein kinase B; JNK: Jun N-terminal kinase; BCL-2: B-cell lymphoma 2.

GLP-1R agonism mediates the inhibition of β-cell apoptosis through PI-3K and PKA pathways. PI-3K/Akt-PKB activates the nuclear transcription factor nuclear factor κβ (NF-κB) and up-regulates its target genes, antiapoptotic proteins B-cell lymphoma 2 (bcl-2) and inhibitor of apoptosis protein 2 (IAP2). Akt-PKB suppresses caspase-3 activation by inhibiting the p38 MAPK/Jun N-terminal kinase (JNK) pathway, a mechanism also involved in endoplasmic reticulum (ER) stress reduction. PKA pathway includes the complex between phosphorylated nuclear cAMP response element binding protein (CREB) and dephosphorylated transducer of regulated CREB activity 2 (TORC2) that up-regulates the expression of the anti-apoptotic gene bcl-2. PKA activation also leads to caspase-3 inhibition [9]. Moreover, GLP-1 improves β-cell function and survival via endoplasmic reticulum (ER) stress reduction, a mechanism involving Activating Transcription Factor 4 (ATF-4) up-regulation [11]. An overview of these signaling cascades is comprised in Figure 1. 

Pro-inflammatory cytokines have been involved in β-cell dysfunction, leading to β-cell apoptosis and alteration in glucose-mediated insulin secretion. In an experimental study, the co-incubation of GLP-1 with cytokine-treated human islet cells protected against β-cell dysfunction. GLP-1 mediated the expression profile of proteins involved in cytoskeleton composition, chaperons, metabolic pathways, and islet regeneration, suggesting that GLP-1 has cytoprotective effects regarding protein networks in the inflammatory dysfunction of islet cells [30]. 

### 2.2. GIP 

After meal ingestion, GIP is secreted from intestinal enteroendocrine K cells, which are mainly found in the duodenum and proximal small intestine, as a final product of cleaved proGIP. PC 1/3 produces the most prevalent form of GIP, GIP(1–42), whereas PC2 produces the shorter form, GIP(1–30)NH2. GIP is also metabolized by the DPP-4 enzyme, but unlike GLP-1, GIP is not degraded in the capillaries of the intestinal mucosa and only approximately half of the circulating GIP is degraded by the plasmatic DPP-4 [18]. Intestinal K cells can be activated by all macronutrients to stimulate the secretion of GIP. Carbohydrates act on the SGLT1 receptors on the apical surface of K cells. The coupled glucose and sodium uptake into the cells activate voltage-gated calcium channels, resulting in increased cytosolic calcium that triggers GIP secretion. Lipids increase the secretion of GIP by activating G-protein-coupled receptors for long-chain free fatty acids (GPR40/FFAR1 and GPR120/FFAR4) expressed on K cells. The mechanisms involved in the stimulation of K cells by amino acids include electrogenic H^+^ or Na^+^ -dependent transporters and G-protein coupled receptors [31]. 

GIP binds to its receptors (GIPR) in pancreatic β-cells. GIPR activation in the pancreas increases cAMP levels and further activates PKA/MAPK and PI-3K/PKB pathways involved in glucose-dependent insulin secretion and biosynthesis. PKA activates MAPK and CREB and produces an intracellular accumulation of calcium involved in insulin granule exocytosis. PI-3 K phosphorylates PKC and PKB causing the subsequent activation of NF-κβ [32]. The increase in insulin secretion is the result of cAMP activation, K-ATP channel inhibitions, intracellular Ca^2+^ increase, and insulin granule exocytosis. GIPR activation mediates the same pathways as GLP-1R (Figure 1) and improves β-cell function and survival via cytoprotective effects as depicted in Figure 1 [9,11]. An observed difference between survival pathways activated by incretins is that PI-3K/Akt-PKB cascade includes the phosphorylation and nuclear expulsion of forkhead transcription factor (Foxo1). Foxo1 is a nuclear transcription factor that down-regulates the pro-apoptotic gene bax involved in glucolipotoxicity-mediated apoptosis of islet β-cells [9]. Campbell JE and colleagues showed that GIPR control on β-cell function and survival involves the expression of T-cell-specific transcription factor-1 (TCF1), a protein responsible for cytoprotection and mediation of insulin secretion, in a cAMP-independent and extracellular signal-regulated kinase (ERK)-dependent pathway. TCF-1 targets the expression of pituitary tumor-transforming gene 1 (PTTG1), which encodes securin, a protein responsible for chromosome stability and DNA repair. The authors concluded that GIPR mediation of TCF-1/PTTG1 expression is a supplementary mechanism of insulin secretion and survival and β-cell adaptation to metabolic stress [33]. 

GIPRs are mainly found in the pancreas, but other extra-pancreatic tissues, such as the gut, adipose tissue, cardiomyocytes, endothelial cells, and central nervous system, express GIPR on the surface of the cells. GIPR activation has been associated with multiple effects in cardiac and vascular tissue such as a decrease in cardiac triglyceride content by increasing phosphorylated hormone-sensitive lipase (P-HSL) and fatty acid (FA) oxidation and increase in both vasoconstrictors and vasodilators agents, such as NO and ET-1 [32]. It has been observed that the extra-pancreatic effects involve a complex interplay between the direct GIPR activity in the peripheral tissue and the secondary effect of GIPR-mediated insulin secretion in β cells.

- Ghrelin

Ghrelin is a peptide formed of 28 amino acid residues that are secreted mainly from the A-like gastric oxyntic cells, but small amounts can be found in the intestinal cells. It has been discovered as a stimulator of growth hormone (GH) release that binds to growth hormone secretagogue-receptor (GHS-R). The chemical structure of human active ghrelin consists of an acetylated third serine residue with an eight-carbon fatty acid, octanoate, essential for the binding to GHS-R and the endocrine effects [34]. The enzyme responsible for the activation of ghrelin is GOAT (Ghrelin O-Acyltransferase), a hydrophobic enzyme bound to the membrane of gastric and intestinal cells, the main ghrelin-secreting tissues [35]. The ghrelin system comprises ghrelin-related peptides that are generated by the alternative splicing of the ghrelin gene that encodes the precursor named preproghrelin, or in the post-translational stage and the pathways involved activated upon specific receptor binding. Preproghrelin contains a 23-amino acid signal peptide and proghrelin, which undergoes proteolytic cleavage and generates the mature ghrelin peptide and an additional C-terminal ghrelin. C-ghrelin can undergo further proteolysis and generate obestatin, a functional peptide [12]. 

The human gene of GHS-R encodes two splicing variants, GHS-R1a, the active receptor, and GSH-R1b, considered functionally inactive. GHS-R1a activates different second messengers, depending on the cell subtype. It has been associated with G protein signaling pathways: phospholipase C/inositol phosphate production and calcium mobilization and adenylate cyclase/PKA and N-type Ca2+ channels opening. However, both GHS-R1a and GHS-R1b can form heterodimers with other G-protein coupled receptors and have different functions from their components [12]. The analysis of the molecular properties of a GHSR-1a agonist, using computational studies, such as homology modeling, molecular docking, and molecular dynamic simulations, suggested that the binding to the GHS-R1a involves hydrophobic interactions between residues of the N-terminal segment and the hydrophobic sub-pockets of the receptor, with possible charge–charge interactions serving as an anchor for the binding [36]. Des-acyl ghrelin, the unacylated form, and the predominant plasmatic form does not bind to GHR-S, and it is not involved in GH secretion but has been associated with specific effects in the peripheral tissue, including cardiovascular actions, through different proposed receptors, such as ghrelin receptor-like receptors or unacylated ghrelin receptors [37]. In an in vitro study, both ghrelin and des-acyl ghrelin showed antiapoptotic effects on the endothelial cells and cardiomyocytes through activation of a different but undiscovered receptor that further stimulates ERK 1/2 and PI3K/Akt pathways [38]. 

The ghrelin system manifests both endocrine properties, such as regulation of pituitary hormones and pancreas secretion, and non-endocrine actions, such as control of energy balance, stimulation of appetite, secretion of gastric acid, gastrointestinal peristalsis, β-cell survival, adipogenesis, neuroprotection, immune regulation, and cardiovascular modulation [12]. Peripheral secreted ghrelin controls gastrointestinal functions via a complex circuit that relies on receptors found in the vagal nerve endings in the stomach, vagal afferent pathways, central mechanisms relying on noradrenaline signaling in the solitary nucleus, and efferent autonomic nervous system [39].

- Peptide YY

Peptide tyrosine–tyrosine, so-called PYY due to its tyrosine residues (Y being the symbol for tyrosine), is a gut hormone with a protein structure consisting of 36 amino acids discovered in 1982 by isolation from porcine upper-intestinal wall [40]. Together with Pancreatic Polypeptide (PP) and Neuropeptide Y (NPY), it forms the NPY family of peptides. PYY is mainly secreted by the L-cells of the distal gut-colonic and rectal cells in postprandial conditions (especially fat consumption) along GLP-1, but there is evidence that it can be also secreted in the nervous system and other organs, such as pancreatic alpha, delta, and PP cells [14]. After secretion, it is rapidly degraded to PYY_3–36_ by the same enzyme that acts upon GLP-1 and NPY (but not on PP), the ubiquitous dipeptidyl-peptidase-4 (DPP-4), by the removal of N-terminal Tyr^1^-Pro^2^ [41]. This modification enables PYY to link to some types of receptors (such as Y2 or Y5) but does not allow it to bind to other types of receptors that require intact structure, such as Y1. Thus, the DPP-4 enzyme rather modulates the biological activity of PYY, but it does not inactivate the hormone, as in the case of GLP-1 [42]. In the process of proteolytic cleavage by DPP-4, PYY turns into PYY_3–36_, the major circulating form. PYY is secreted as a response to the presence of nutrients in the distal gastrointestinal tract, with a maximum level after 1–2 h postprandially, being an important regulator of food intake, most known as an anorectic peptide [13]. 

PYY is an important regulator of glucose metabolism, with divergent functions of PYY_1–36_ and PYY _3–36_. Pancreatic PYY has a direct inhibitory effect on insulin release, whereas circulating PYY_3–36_ improves glucose tolerance and insulin secretion partly through the activation of GLP-1 secretion from L cells and regulation of insulin sensitivity [15]. PYY increases β-cell proliferation and protects against β-cell apoptosis via the activation of Y1 receptors, Y4 receptors, and Y5- receptors in humans [13,14]. In a study using protein structure prediction, molecular docking stimulation, and molecular dynamics simulation, the conserved amidated C-terminal part of PYY showed a greater affinity for the Y4 receptor than the Y1 receptor. The formation of the protein complex between PYY and Y receptors is based mainly on non-polar interactions [43].

In the gut, PYY is involved in the ileal break induction and inhibition of epithelial electrolyte secretion, favoring nutrient absorption, via basolateral Y1 and Y2 receptors in a cAMP-dependent-Cl-secretion mechanism. Moreover, PYY signaling mediates gut microbiome composition. PYY deficiency altered the composition of the bacteria phylum in the gut in high-fat diet conditions and caused alterations in the expression of tight junction proteins [44]. Regarding the cardiac system, PYY was associated with cardiovascular risk factors and major acute cardiovascular events as well as cardiac mortality during follow-up in patients presenting with acute coronary syndromes, although the association was lost after adjusting for multiple confounders [45].

- Neurotensin

Neurotensin (NT) is released as a 13-aminoacid peptide from the neuroendocrine cells found in the gastrointestinal tract and central nervous system in a calcium-dependent mechanism after the cleavage of an inactive precursor of 169 amino acids. NT activates the family of NT receptors (NTR), which comprises three subtypes of receptors, NTS1, NTS2, and NTS3, upon recognition of the C-terminal 8–13 sequence. NTS1 and NTS2 are G-protein coupled receptors and NTS3 are intracellular receptors [46]. Intraluminal content of nutrients and pancreatic juice and bile promote NT secretion and regulate the amount of NT [16]. NT regulates many gastrointestinal processes. NT facilitates the absorption of nutrients, particularly lipids, via an increase in the local blood flow and stimulation of the pancreatic and hepatic bile acid secretion, increases fluid and electrolyte secretion, mediates glucose-sensitive insulin secretion, inhibits the apoptosis of pancreatic β cells, controls gut motility in a multilevel manner that includes enteric and central nervous system and other enteric hormones, and has anorectic properties regarding appetite control. NT controls caloric intake via a negative feedback loop, where the acute increase in NT produced by the lipid’s ingestion inhibits further caloric intake at the hypothalamic level. The interaction between NT and mast cells via NTR1 is involved in the enteric protection including enteric inflammation and carcinogenesis and regulation of gut microbiome composition and function. However, NT also proved to exert entero-protective effects via anti-oxidative, anti-inflammatory and antiapoptotic properties [16]. In the cardiovascular system, NT was associated with improved myocardial performance and increased contractility, divergent effects over blood pressure control, depending on the type of experimental study, and different regional vascular effects [46]. Elevated pro-neurotensin levels, a precursor of NT, predicted the development of metabolic disturbances related to cardiovascular risk [47] and were associated with a 10-year very high cardiovascular risk in type 1 diabetic patients [48]. NT levels post-PCI have been associated, in a U shape manner, with adverse outcomes of major acute cardiovascular events during long-term follow-up, particularly in female patients undergoing coronary stenting [49].

- CCK

Cholecystokinin (CCK) is a gut peptide secreted from the endocrine I-cells and a member of the family of ligands of CCK1 and CCK2 receptors. CCK peptides manifest their biological functions via binding to the specific receptors upon the recognition of the conserved C-terminal heptapeptide sequence. PreproCCK is the primary translational product which is formed of 115 amino acids, with the following cleavage processions being cell-specific: CCK peptides are secreted in CCK-58, -33, -22, and -8 forms; CCK secretion is induced as a response to the gut lumen rich in nutrients, such as proteins and fat; the stimulation of the CCK1 or CCK-A receptor drives the gastrointestinal functions responsible for the release of bile and pancreatic secretions, control of gastrointestinal motility, delay of gastric emptying and inhibition of gastric acid secretion, whereas CCK-B or CCK2 receptor is the major receptor from the brain [17]. 

The cardiac form of CCK is a cleaved peptide that is lacking the N-terminal domain and is triple-sulphated, which is different from intestinal and cerebral forms [50]. ProCCK is involved in heart development via transcription factors TBX5 and MEF2C. ProCCK showed dynamic transcriptional changes related to heart dysfunction as induced by myocardial infarction, mechanical stretch, or endothelin 1 [51].

- Gut microbiota biomarkers: a gut metabolism hypothesis

Gut microbiota consists of all the microorganisms that coexist in a well-established balance at the intestinal level. Gut microbiota regulates local and systemic functions, interfering with digestive, immune, and metabolic effects via the secretion of gut microbiota-derived components. The disturbances of gut microbiota composition, known as dysbiosis, can alter the release of microbial products that activate metabolic pathophysiological processes, defining a gut metabolism hypothesis: dysbiosis and microbial metabolites are involved in metabolic disorders, such as diabetes mellitus and dyslipidemia, through both a direct metabolic effect and the enhancement of pro-inflammatory signaling pathways [52]. Moreover, the interaction between the host genome, microbial metagenome, and diet correlates with cardiometabolic risk factors and influences the development of atherosclerotic cardiovascular diseases. In a study based on twins, the composition of fecal microbiota was associated with total and visceral fat. The obesity-associated host genetic variants confirmed the heritability estimation for adiposity measures, suggesting that host genes may modulate the relationship between the gut microbiome and metabolic syndrome [53]. Asnicar et al. published a study named PREDICTION 1 regarding the diet–metabolic–microbial metagenome signature and demonstrated a strong link between the microbiome composition, host metabolism, and habitual diet, thus concluding that a. the composition and diversity of microbiome are related to diet, b. the habitual diet has a strong impact on the composition of the gut microbiome, c. the microbial indicators of obesity are reproducible, d. there are cardiometabolic markers correlated with a specific structure of the gut microbiome, and e. gut microbiome is a predictor for postprandial TG and insulin concentration. In the future, these data may be used for the characterization of the gut microbiome as a biomarker for cardiometabolic risk [54].

- TMAO

Choline and other choline-containing nutrients, such as L-carnitine and betaine, are the precursors of Trimethylamine N-oxide (TMAO). Gut microbiota enzymes metabolize these precursors to TMA, which is further absorbed and transferred to the liver. The hepatic flavin-containing monooxygenase (FMO) family, mostly FMO3, converts TMA into TMAO. Farnesoid X receptor (FXR), a nuclear receptor activated by bile acids, can modulate FMO3 activity [55]. TMAO is an important pro-inflammatory stimulus in systemic circulation that may aggravate metabolic disturbances. A meta-analysis showed a positive dose-dependent correlation between serum TMAO levels and an increased risk of diabetes mellitus development [56]. TMAO was also correlated with disturbances in lipid metabolism, acting on multiple sites, such as the liver and the intestine [57]. Dietary supplementations with TMAO reduced the bile acid pool size, down-regulated the expression of proteins involved in the synthesis and transport of bile acids, and decreased the hepatic expression of *cyp7a1*, a rate-limiting enzyme responsible for the catabolism of cholesterol and bile acid synthesis and *cyp27a1*. In the intestine, TMAO administration reduced cholesterol absorption and decreased the expression of Niemann–Pick C1-like1 (Npc1L1) protein involved in the transport of cholesterol into enterocytes from the gut lumen, and adenosine triphosphate-binding cassette (ABC) G5/G8 protein, which transports cholesterol in a reverse way [58]. TMAO supplementation induced hepatic triglycerides accumulation and steatosis via up-regulation of the lipogenic genes in both in vitro and in vivo models [57].

- LPS

Lipopolysaccharide (LPS), structurally composed of carbohydrates and lipid A, is a component of the outer membrane of Gram-negative bacteria. In the immunity system, LPS is a pathogen-associated molecular pattern (PAMP) that activates the Toll-like receptors (TLR) family, particularly TLR4. Lipid A, the immunogenic part of the LPS molecule, binds to TLR4 and its accessory protein myeloid differentiation protein 2 (MD-2) via CD14, a co-receptor found in the membrane, and recruits the adaptor protein myeloid differentiation primary response protein 88 (MyD88) upon activation of NF-κB. The computational studies and molecular modeling strategies have characterized the process of ligand recognition of TLR4/MD-2 and dimerization mechanisms, the study of mutant TLR4 and MD-2 proteins, the TLR4 modulators, including LPS and non-LPS-like agents, and the intracellular domain of TLR4 [59].

In the circulation, LPS combines with the LPS-binding protein (LBP), a glycoprotein that interacts with lipoproteins for the plasmatic clearance of LPS. In the liver, LPS undergoes enzymatic degradation or bile excretion [60]. When the circulating serum concentration of LPS is above 20 ng/mL, the term low-grade endotoxemia is used. Low-grade endotoxemia reflects gut dysbiosis and the dysfunctional gut barrier. The altered composition of gut microbiota accentuates LPS synthesis and affects the tight junction proteins, enabling LPS transfer into the blood. LPS, via TLR4, activates many pro-inflammatory cascades based on NF-κB-driven signals and recruits inflammatory cells, creating a chronic pro-inflammatory state which increases the cardiometabolic risk [60]. 

LPS and LBP levels are increased in diabetic patients compared to healthy subjects and are exacerbated in the disease onset and advanced complications [61]. LPS alters pancreatic β-cell properties through activated inflammation upon binding to the pancreatic TLR4, which inhibits insulin synthesis. Moreover, in the liver, LPS promotes inflammation and oxidative injury and disrupts insulin signaling, altering lipid and glucose metabolism [62]. Chronic experimental metabolic endotoxemia produced by the LPS infusion induced obesity, diabetes mellitus, and increased hepatic triglyceride content in rodents [63]. LPS showed additional effects regarding lipid metabolism. In animal experimental studies, LPS administration decreased the hepatic expression of genes involved in lipogenesis, such as fatty acid synthase (FAS), Acetyl-CoA carboxylase-1 (ACC-1), and Stearoyl-CoA desaturase-1 (SCD-1) [64], and down-regulated the lipid metabolism-related genes within the white adipose tissue, such as ACC-1, FAS, SCD-1, uncoupling protein 2 (UCP2), and 11 β-hydroxysteroid dehydrogenase type 1 (11β-HSD1) [65]. LPS supplementation induced lipid accumulation in the hepatic cells and favored the expression of the adipose differentiation-related protein (ADRP) in hepatocytes through the inhibition of fatty acid oxidation via down-regulation of peroxisome proliferator-activated receptor (PPAR) α [66].

## 3. Gut Molecules and Cardiovascular Diseases 

### 3.1. Atherosclerosis

#### 3.1.1. Gut Peptides

- GLP-1. GIP

Atherogenesis is a sequential process that involves many molecules and cells that interfere bidirectionally. Lesion initiation and growth involve the activation of endothelial cells (ECs) that cause the recruitment of monocytes, macrophage differentiation, and foam cell development. Advanced lesions include several additional mechanisms: smooth muscle cell (SMC) transition from a contractile state into a proliferative one, SMC migration into the intima and secretion of an extracellular matrix to form a fibrous cap, T and B cell activation induced by antigens in the atherosclerotic lesion and lesions calcification. Atherosclerotic plaques contain a large amount of cholesterol and a necrotic core. The necrotic core consists of foam cells, collagen, and SMC and is the result of the dysfunctional macrophages’ death clearance mechanisms, such as apoptosis–efferocytosis, necrosis, ferroptosis, autophagy, and pyroptosis. Vulnerable atherosclerotic plaques, prone to rupture, are characterized by degradation of the plaque-stabilizing extracellular matrix, reducing the fibrous cap thickness, neoangiogenesis, intraplaque hemorrhage, and activation of inflammatory mechanisms [67]. 

**Figure 2:**
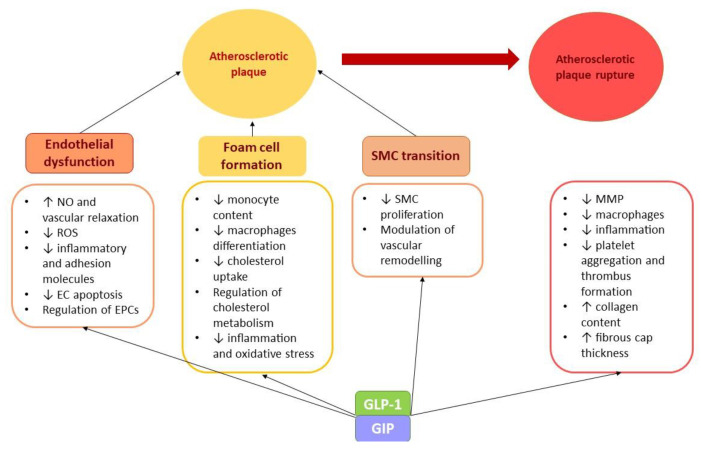
The effects of incretins in atherogenesis. NO: nitric oxide; ROS: reactive oxygen species; EC: endothelial cells; SMC: smooth muscle cells; MMP: metalloproteinase; EPCs: endothelial progenitor cells.

GLP-1, GIP, and incretins-based therapies may improve the atherosclerotic burden through the regulation of steps involved in atherosclerosis progression. In animal studies, GLP-1 and GIP have been associated with improved endothelial function, reduced atherosclerotic lesions formation by decreasing local inflammation and lipids infiltration, and a stable phenotype of the atherosclerotic plaque [68]. The main processes in atherosclerotic plaque development mediated by incretins are summarized in Figure 2. 

Endothelial dysfunction involves complex pathways and is often regarded as the first step in atherosclerosis development. In diabetic patients, high levels of plasmatic glucose over time can alter the endothelium properties. GLP-1 improves endothelial function via increasing nitric oxide (NO), enhancing vasorelaxation, and decreasing reactive oxygen species (ROS) generated during oxidative stress and adhesion molecules induced in hyperglycemic states in the vascular wall [69]. Higher levels of NO occur due to the activation of endothelial nitric oxide synthase (eNOS) expression in cAMP-dependent manner in hyperglycemia conditions [70]. GLP-1 attenuated the endothelial dysfunction, the excessively stimulated autophagy of endothelial cells, and decreased the levels of ROS via reducing the phosphorylation of ERK 1/2 and restoring the expression of epigenetic factor histone deacetylase 6 (HDAC6), a key regulator of redox processes regulation [71]. GLP-1 infusion in cardiac microvascular endothelial cells cultured in high glucose conditions decreased oxidative stress generation and reduced the apoptosis index, improving vascular dysfunction in a cAMP/PKA-dependent manner [72]. GLP-1 was demonstrated to be an important regulator of endothelial progenitor cells (EPCs), which are part of the restoration process of endothelial integrity after an injury, enhancing EPCs proliferation and differentiation via vascular endothelial growth factor (VEGF) generation [73]. In an experimental study based on TNF-α stimulation in endothelial cells, exendin-4, a GLP-1R agonist, downregulated NF-κB activity and adhesion molecules expression, indicating an anti-inflammatory role of GLP-1 in endothelial cells [74]. 

Nagashima and colleagues demonstrated that four-week administration of active forms of GLP-1, GLP-1(7–36) amide, or GIP, GIP(1–42), via binding to its specific receptors in Apo E -/- mice suppresses atherosclerotic lesion initiation and progression through the decrease in macrophages infiltration, foam cell formation and regulation of cholesterol metabolism in macrophages. Incretin treatment reduced the accumulation of cholesterol in macrophages and consecutive foam cell formation induced by oxidized LDL (ox-LDL) and suppressed the expression of adhesion molecules in endothelial cells and the proliferation of smooth muscle cells [75]. GLP-1(7–37) and the split products, GLP-1(9–37) produced by DPP-4 and GLP-1(28–37) produced by neutral endopeptidase-dependent cleavage proved additional anti-atherosclerotic properties involving plaque inflammation and stability. The plaque macrophage infiltration and matrix metalloproteinases-9 (MMP-9) expression were reduced by the GLP-1 products, whereas the plaque collagen content and fibrous cap thickness were increased. Both split products manifested similar properties with the GLP-1(7–37), although neither of them acted on GLP-1 classic receptors, so it was suggested that other supplementary mechanisms could be involved in the observed atheroprotective effects of GLP-1 [76]. 

GLP-1 protects against vascular remodeling through inhibition of vascular smooth muscle cell migration and proliferation, a mechanism involving increased mitochondrial activity and mitochondrial fusion and inhibition of dynamin-related protein 1 (DRP1) in a PKA-dependent manner [77]. In an in vitro study over coronary smooth muscle cells stimulated by TNF-α in the context of atherosclerosis, the authors concluded that exendin-4 downregulates the expression of MMPs through inhibition of Akt signaling pathway, suggesting an important role for GLP-1 in atherosclerotic plaque vulnerability [78]. 

In diabetic Apo E -/- mice, long-term GIP infusion caused a reduction in LDL-induced foam cell production and macrophage-driven atherosclerotic lesions, even though GIPR expression in macrophages was mildly reduced by the diabetic state [79]. The inhibition of foam cell formation by macrophages was based on the suppression of cyclin-dependent kinase 5 (Cdk5)/CD36 pathway, a pathway that has been correlated with LPS-induced inflammation and advanced glycation end products (AGEs)-derived atherosclerosis [80]. In an experimental study, GIP overexpression improved atherosclerotic plaque stability by decreasing macrophage infiltration and increasing collagen content. Treatment with GIP reduced monocyte migration and prevented pro-inflammatory signaling pathways in macrophages activated by endotoxin [81]. 

On the other hand, the authors of one study obtained unexpected results regarding the GIP effects on vascular cells. GIP induced the expression of osteopontin, a proatherogenic cytokine, and produced the release of endothelin-1 and activation of CREB in the mouse arterial wall via but without engaging the traditional cAMP/PKA pathway. CREB was involved in the mediation of GIP-stimulated ET-1 release in endothelial cells and the regulation of ET-1-activated osteopontin expression in vascular smooth muscle cells. GIPR expression and osteopontin mRNA levels from human carotid endarterectomy sections were positively correlated with the number of clinical events and some characteristics of unstable atherosclerotic plaques, such as the extent of lipid accumulation, macrophage infiltration, elastin content, and pro-inflammatory molecules. This unexpected association suggests that GIPR expression manifests plasticity related to the vascular phenotype in the context of atherosclerosis. Moreover, the increased risk of vascular events, such as stroke, in patients with type 2 diabetes, was associated with the A allele of the common variant rs10423928 of GIPR gene [82]. Following this observation, two prospective studies of independent populations, Malmö Diet Cancer–Cardiovascular Cohort (MDC-CC) and Prevalence, Prediction, and Prevention of Diabetes in Botnia (PPP-Botnia), have demonstrated that high fasting levels of GIP, but not GLP-1, are associated with an increased risk for coronary artery disease, myocardial infarction, and cardiovascular and all-cause death [83]. In participants of MDC-CC at re-examination, GIP and GLP-1 fasting levels proved to be divergent in association with markers of subclinical atherosclerosis: increased fasting GIP levels were associated with increased intima-media thickness in the common carotid artery and in the carotid bifurcation, while GLP-1 was associated with decreased intima-media thickness in the carotid bifurcation [84]. 

DPP-4 inhibitors were reported to have suppressive effects on atherosclerosis development and progression as a response to increased serum levels of GIP and GLP-1. Terasaki M and colleagues showed that administration of vildagliptin, a DPP-4 inhibitor, attenuates atherosclerotic lesion progression and reduced macrophage accumulation and foam cell formation, effects based partially on GIP and GLP-1 pathways. The study also proved that vildagliptin confers atheroprotective effects beyond that of incretins [85]. In a review of preclinical and clinical studies, vildagliptin was associated with vasculo-protective effects: suppression of inflammation and leukocyte adhesion, regulation of lipid metabolism, mediation of vascular tonus, improvement in endothelial dysfunction and antithrombotic properties [86]. 

Human studies, despite some controversial effects regarding the incretin’s effects in subclinical atherosclerosis, have shown a significant reduction in cardiovascular events using GLP-1 agonists in patients with diabetes mellitus type 2. GLP-1 infusion during hyperglycemia conditions protected against endothelial dysfunction and oxidative stress generated by high serum concentration of glucose, an effect dependent on the level of glycemia [87]. In a randomized study, in sixty-nine patients with type 2 diabetes, treatment with exenatide for 51 weeks resulted in a significant decrease in oxidative stress markers, malondialdehyde (MDA), oxidized low-density lipoprotein (ox-LDL), and postprandial glycemia and lipidaemia as compared to insulin treatment [88]. In a randomized-controlled trial, exenatide treatment for 52 weeks reduced subclinical atherosclerosis, as assessed by the carotid-intima media thickness (CIMT), and lipid metabolism markers in patients with type 2 diabetes mellitus [89]. A meta-analysis of randomized placebo-controlled cardiovascular outcome trials assessing GLP-1 receptor agonists in patients with type 2 diabetes mellitus, including 56,004 patients with and without established cardiovascular disease, showed a 12% decrease in the primary composite endpoint regarding major adverse cardiovascular events, including cardiovascular mortality, non-fatal MI and non-fatal stroke. Treatment with GLP-1 R agonists reduced the risk of CV and all-cause mortality, fatal and non-fatal stroke, and heart failure hospitalization [90]. A possible mechanism responsible for this observation is the reduction in atherothrombotic events by the GLP-1 agonists. In an in vitro and ex vivo study, exenatide inhibited platelet aggregation induced by thrombin, ADP, or collagen and reduced the thrombus growth, effects based on the GLP-1R signaling [91]. DPP-4 inhibition and the former substrate, the intact form of GLP-1, GLP-1(7–36), also suppressed platelet aggregation and thrombus expansion under physiological flow conditions, but without the involvement of GLP-1R on the platelets [92].

- Ghrelin

Ghrelin demonstrated positive effects regarding the cardiovascular atherosclerotic disease. In a long-term 19-year follow-up, high ghrelin plasmatic concentrations were associated with protection against coronary heart disease and the C/C variant of the ghrelin promoter was a protective variant in hypertensive subjects [93]. Des-acylated ghrelin was negatively associated with subclinical atherosclerosis in middle-aged women with metabolic syndrome, as assessed by carotid artery intima-media thickness, suggesting gender-specific effects of the des-acylated ghrelin in the development of atherosclerosis [94].

In experimental studies, ghrelin exhibited atheroprotective properties: inhibition of endoplasmic reticulum stress in endothelial cells [95], stimulation of NO synthase in the endothelium via PI3k/Akt/eNOS pathway [96], and consecutive improvement in the endothelial function [97], a decrease in inflammatory reaction and oxidative stress induced by oxLDL [98], attenuation of vascular calcification in a 5’ adenosine monophosphate-activated protein kinase (AMPK)-dependent manner and consecutive autophagy upregulation [99]. Moreover, unacylated ghrelin revealed anti-atherosclerotic effects through regulation of oxidative stress in endothelial cells and decrease in adhesion molecules and inflammatory cells via overexpression of miR-126, an important regulator of vascular inflammation, as well as induction of superoxide dismutase-2 (SOD-2) and mediation of sirtuin 1 (SIRT1) expression, defenders of oxidative stress injury [100], and inhibition of lipid accumulation within the vascular wall [101]. 

The uncontrolled inflammatory reaction and pro-atherogenic processes increase the risk of plaque rupture and thrombosis. In an animal model of atherosclerosis, ghrelin treatment inhibited atherosclerosis progression and increased the stability of the atherosclerotic plaque via a decrease in neo-vessel formation, MMPs activity, macrophage content, and inflammation markers within the lesion [102]. Ghrelin receptor (GHS-R1) deficiency produced an unstable phenotype of the atherosclerotic lesion, increasing the expression of adhesion molecules and cytokines and reducing the smooth muscle cell content [103]. 

Following the beneficial observed effects in atherosclerosis development and progression, ghrelin was suggested to exert protective action against in-stent restenosis. The described mechanisms were similar to the anti-atherosclerotic properties: reduction in inflammation, inhibition of vascular smooth muscle cell proliferation and migration in a cAMP/PKA dependent manner, amelioration of endothelial dysfunction via endothelial cell proliferation and eNOS modulation, and inhibition of platelet aggregation and thrombosis [104]. 

Ghrelin has additional roles in improving angiogenesis in ischemic conditions. In a MI model in diabetic rats, ghrelin improved left ventricular contractility and microvascular density reduced the infarct size, and ameliorated angiogenesis, through GHS-R1a-mediated AMPK/eNOS signaling pathways and upregulation of vascular endothelial growth factor (VEGF), Hypoxia-inducible factor 1-alpha (HIF1-α) and its receptors [105]. In a mouse model of critical limb ischemia, ghrelin promoted the generation of new capillaries and arterioles, with results based on the reduction in apoptosis and fibrosis via the activation of pro-survival Akt/VEGF/Bcl-2 signaling pathways. The genetic mechanisms were the up-regulation of proangiogenic microRNAs (miR-126 and miR-132) and antifibrotic microRNAs (miR-30a) and the down-regulation of antiangiogenic miRNAs (miR-92a and miR-206) induced by ghrelin [106]. 

#### 3.1.2. Gut Microbiota

- TMAO

Intestinal microbiota plays an important role in atherosclerosis development and progression and modulation of atherosclerotic lesion stability. The intestinal metabolites secreted by the gut microbiota possess different properties that can accelerate or protect against atherosclerosis. 

TMAO is involved in all stages of atherosclerosis development through different molecular mechanisms that affect endothelial function, vascular inflammation and calcification, lipid metabolism, and plaque progression [107]. TMAO and its precursor, choline, showed to be independent predictors of cardiovascular diseases in apparently healthy middle-aged subjects [108]. In human studies, TMAO levels were correlated with markers of early atherosclerosis development, such as an increased carotid intima-media thickness, independent of other cardiovascular risk factors, insulin resistance, obesity, or fatty liver [109]. In a linear multiple regression model, TMAO was an independent predictor of carotid atherosclerotic lesions, even after adjusting for the traditional cardiovascular risk factors [110] and in symptomatic patients with peripheral artery disease, TMAO levels predicted the disease severity and the cardiovascular mortality [111]. A meta-analysis of 19 prospective studies which included patients that underwent coronary angiography or patients diagnosed with heart failure, chronic kidney disease, or diabetes mellitus concluded that elevated serum levels of TMAO and its precursors (L-carnitine, choline, or betaine) are correlated with an increased risk for major acute vascular events and cardiovascular mortality, independent of the traditional cardiovascular risk factors [112]. Moreover, γ-butyro-betaine (γBB), an intermediary metabolite that forms in the process of conversion of carnitine to TMAO [113], has been associated with carotid atherosclerosis and a higher risk for cardiovascular mortality [114]. 

Endothelial dysfunction is a process that affects endothelium properties and structure, often regarded as the first step in atherosclerosis development. TMAO was shown to affect endothelial function through activation of high-mobility group box protein 1 (HMGB1), a pro-inflammatory agent that decreases the expression of proteins involved in cell–cell junctions, resulting in a permeable and dysfunctional endothelium, and further upregulation of TLR4, a pro-inflammatory receptor [115] and pyroptosis of endothelial cells through activation of succinate dehydrogenase complex subunit B (SDHB) that increases ROS levels and impairs mitochondrial function [116]. TMAO decreased the endothelial properties of self-repair after cell injury and increased the monocyte adhesion in a PKC/NF-κB/VCAM-1-dependent manner [117] and activated ROS-thioredoxin-interactive protein (TXNIP) cascade, responsible for the stimulation of NLRP3 inflammasome, the release of pro-inflammatory cytokines and reduction in NO [118]. Moreover, TMAO stimulated the inflammation and oxidative stress reactions in endothelial progenitor cells (EPCs) and showed detrimental effects regarding the functions of tube formation and migration of EPCs in patients with stable angina [119]. 

The stimulation of vascular inflammation by TMAO includes many signaling cascades associated with atherosclerosis development. TMAO induced a pro-inflammatory environment in the vascular wall and favored the expression of IL-1β, IL-6, TNF-α, NF-κB, MMP9, and NLRP3 and activated oxidative stress cascade. The authors of this study demonstrated that oxidative stress activation is required for TMAO-induced inflammation, and it involves molecular mechanisms, such as the suppression of the AMPK/SIRT1 signaling pathway [120]. In an in vitro study, TMAO induced the inflammatory reaction and the synthesis of ROS and favored the expression of adhesion molecules in vascular smooth muscle cells via nicotinamide adenine dinucleotide phosphate oxidase 4 (Nox4)/protein arginine methyltransferase 5 (PRMT5)/NF-κB p65/VCAM-1 signaling cascade [121]. Supplementary studies showed that TMAO could stimulate MAPK/ERK/NF-κB cascade [122] and NLRP3 activation through the SORT3-SOD2-mitochondrial ROS signaling pathway [123] to promote vascular inflammation as an atherogenic condition. 

TMAO administration in mice increased the atherosclerotic burden and the lipid content in plasma and altered the bile acid profile. TMAO inhibited bile acid synthesis through activation of small heterodimer partner (SHP) and farnesoid X receptor (FXR) pathway and downregulation of Cyp7a1 expression, suggesting that TMAO-induced atherosclerosis progression is correlated with disturbances in lipid and bile acid metabolism [124]. In an animal model of atherosclerosis, TMAO promoted the formation of foam cells and the progression of atherosclerosis, enhancing macrophage recruitment, and the expression of pro-inflammatory cytokines and adhesion molecules via the CD36/MAPK/JNK signaling pathway [125]. The formation of foam cells in the vascular wall includes a series of molecular events that require macrophage receptors and cholesterol particles. TMAO showed pro-atherogenic effects in the foam cell formation: upregulation of macrophage scavenger receptors, downregulation of exporters involved in reverse cholesterol transport [126], and alteration of the electrostatic properties of the interface between endothelial cells and vascular lumen, affecting the influx/efflux of the cholesterol droplets [127]. Luo T and colleagues showed that TMAO may favor coronary artery disease development, even in well-controlled LDL-c levels, by inducing cholesterol accumulation and vascular inflammation via reducing proline/serine-rich coiled-coil protein 1 (PSRC1) expression. PSRC1 demonstrated anti-atherogenic properties, including the stimulation of reverse cholesterol transport and inhibition of inflammation [128].

Vascular calcification is a process affecting mainly the vascular smooth muscle cells that characterize advanced atherosclerotic lesions. TMAO has been involved in the process of vascular calcification and consecutive atherosclerosis progression. In an in vitro, in vivo, and ex vivo study, TMAO promoted the calcification of smooth muscle cells via activation of genes that regulate osteogenic differentiation, such as Runx2 (Runt-related transcription factor 2) and BMP2 (bone morphogenetic protein-2), and stimulation of NLRP3 inflammasome and NF-κB [129].

In a tandem stenosis animal model, TMAO levels were associated with properties of vulnerable atherosclerotic plaques, such as intraplaque hemorrhage, and markers of inflammation and platelet activation. In the same study, TMAO levels, increased in both healthy and unhealthy diets, were not correlated with the extent of atherosclerosis in either prone to atherosclerosis animal studies or Framingham Heart Study [130]. One possible explanation for the association between TMAO levels and the instability of the atherosclerotic plaque is the impairment of M2 polarization and efferocytosis of macrophagocytes induced by TMAO [131]. TMAO was described as a marker of plaque instability and a predictor of plaque rupture vs. erosion as assessed by OCT in STEMI patients [132] and was correlated with the severity of coronary atherosclerosis in patients presenting with acute coronary syndrome [133]. The pro-thrombotic activity of TMAO occurs due to the increase in calcium release from intracellular stores and enhancement of the platelet activation and responsiveness to different pro-coagulant factors, and consecutive thrombosis potential [134] via phosphorylation of ERK1/2/JNK in platelets [135]. In STEMI patients undergoing PCI, TMAO levels fluctuated during the 4-month follow-up and these changes predicted the infarct size, showing significant association, particularly in patients with impaired renal function, suggesting that TMAO could be involved in the ventricular remodeling [136]. 

The gut≥–artery interaction proposes additional epigenetic mechanisms involved in TMAO signaling. The authors of one in vitro study suggested that TMAO association with atherosclerosis is related to the modulation of miR-17/92 cluster induced by TMAO, which is responsible for the up-regulation of genes involved in inflammation and pro-thrombotic activity, such as IL-12A and PAI-1, plasminogen activator inhibitor 1 [137]. TMAO was associated with the overexpression of miR-21-5p, a genetic factor related to inflammation, and miR-30c-5p, involved in cholesterol and fatty acid metabolism. The expression of PER2, a target gene of both miR-21 and miR-30c and a circadian rhythm regulator, was decreased after TMAO administration, a disruption that can lead to increased cardiovascular risk [138]. In an animal model of TMAO-driven atherosclerosis, TMAO levels up-regulated the expression of miR-146a-5p, as a regulator of NF-κB-driven inflammation [139]. TMAO activated hepatocytes to secrete exosomes that are taken up by the vascular endothelial cells and suppressed the endothelial function and angiogenesis through inhibition of C-X-C chemokine receptor type 4 (CXCR4) expression [140]. The exacerbation of atherosclerosis produced by TMAO includes a positive feedback loop generated by the up-regulation of lncRNA enriched abundant transcript 1 (NEAT1), a regulator of endothelial cell behavior, and miR-370-3p/signal transducer and activator of transcription 3 (STAT3)/flavin-containing monooxygenase-3 axis (FMO3), a cascade that produces excessive proliferation and decreased apoptosis of human aortic endothelial cells [141].

- LPS

LPS has been studied as a participant in all steps of atherosclerosis development. In an atherosclerosis-prone animal model, increased LPS levels, as a marker of gut dysbiosis, potentiated the progression of atherosclerotic plaques and promoted the proliferation of vascular smooth muscle cells via osteopontin production by monocytes in an NF-κB dependent manner. Moreover, in patients with carotid atherosclerosis, LPS and osteopontin levels were increased and showed a positive correlation, indicating a relationship between gut dysbiosis and atherogenesis both in humans and animals [142]. In patients with PAD, increased LPS levels were associated with the atherosclerotic burden and markers of oxidative stress activation [143]. Lipoprotein particles carry LPS and 3-hydroxy fatty acids (3OHFAs), the immunogenic part of LPS, by binding to LPS-binding protein. The lipid particles charged with LPS may be transferred into the subendothelial space and produce a pro-inflammatory reaction in the atherosclerotic lesion. Although LPS was detected in all lipoprotein particles, the highest amount was transported by LDL and HDL and the quantity of LPS was higher in VLDL particles, with high variability between the subjects being evidenced [144]. 

In the endothelial cells, LPS showed pro-inflammatory properties and increased miR-146 and CXCL16 expression, a chemokine and adhesion molecule, via TLR4/NF-κB. MiR-146, a negative regulator of atherosclerosis and inflammation, mediated the CXCL16 expression induced by LPS in a TLR4-dependent manner and controlled the inflammation induced by NF-κB through a negative feedback loop [145]. LPS stimulated the expression of adhesion molecules and pro-inflammatory cytokines in senescent endothelial cells, enhancing the basal inflammation state [146] and activated the pro-inflammatory transcription factor NF-κB through Jumonji domain-containing protein D3 (Jmjd3) expression [147], showing complex inflammatory signals involved in the atherogenesis at the endothelial level. 

Macrophages are key players that coordinate inflammation, foam cell formation, smooth muscle cell function, and vascular calcification in atherogenesis. Foam cell formation is dependent on the uptake of LDL and mostly oxidized LDL. LPS activated the expression of scavenger receptors in the macrophages, enhancing the uptake of LDL and foam cell formation in a JAK/STAT-dependent pathway [148]. Another signaling cascade influenced by LPS in the activation of scavenger receptors and following the uptake of LDL was MAPK/ERK. LPS increased the scavenger receptor CD204 expression in a MAPK/ERK-dependent manner, whereas CD36 was activated in an ERK-independent way [149]. The uptake of oxLDL into macrophages through the up-regulation of lectin-like oxLDL receptor-1 (LOX-1) expression was induced by LPS via the ERK1/2 signaling pathway [150]. In an in vitro study, LPS promoted inflammation in the murine macrophages cell line by enhancing the expression of adipophilin, a protein involved in foam cell formation and inflammation, through ERK1/2- peroxisome proliferator-activated receptors (PPAR)-γ pathway [151]. iNOS is a key factor involved in macrophage-driven inflammation that can be up-regulated by LPS via myocardin-related transcription factor A (MRTF-A) by bonding to its promoter [152]. 

Macrophages apoptosis has a dual role in atherogenesis: it confers beneficial effects during the first steps of atherosclerosis development, but in advanced lesions, macrophages apoptosis increases the necrotic core and the instability of the atherosclerotic plaque that becomes more prone to rupture and subsequent thrombosis. LPS produced macrophage apoptosis through sphingosine-1-phosphate (S1P) up-regulation, consecutive decreased urocortin expression, and activation of cytosolic phospholipase A2 (cPLA2) mediated apoptosis pathway. The activation of lipoprotein-associated phospholipase A2 (Lp-PLA2) and p38 and JNK members of the MAPK superfamily by LPS modulated apoptotic cascade [153]. Macrophages stimulated with LPS showed increased levels of IL-1β, IL-6, TNF-α, NLRP3 inflammasome, and ROS as markers of inflammation and oxidative stress and provided the decreased activity of autophagy and biogenesis of extracellular vesicles (EVs). EVs derived from LPS treatment induced inflammation and oxidative stress activation in smooth muscle cells and produced an osteogenic switch of the vascular smooth muscle cells, promoting microvascular calcification, partially due to the decrease in matrix gla protein, an inhibitor of calcification [154]. However, LPS treatment favored the expression of miR-21 in macrophages, a negative regulator of the TLR-4/NF-κB cascade, as a compensatory mechanism for the lipid accumulation and inflammation disturbances induced by LPS [155]. Yu MH and colleagues studied the effect of LPS stimulation in vascular smooth muscle cells and showed that LPS promoted serum amyloid A1 (SAA1) secretion in a concentration and time-dependent manner and activated a pro-inflammatory cascade that includes SAA1-NOX4/ROS-p38MAPK/NF-κB with the release of IL-1β, IL-6, IL-8, IL-17, TNF-α and MCP-1 [156].

### 3.2. Heart Failure

#### 3.2.1. Gut Peptides

- GLP-1. GIP

Heart failure pathophysiology relies on multiple processes affecting the structure and function of the heart, such as cardiac ischemia and subsequent myocardial infarction-induced necrosis, hypertrophy, fibrosis, inflammation, cardiomyocytes apoptosis, and electrolyte imbalance causing disturbances in cardiac electrolyte handling, particularly calcium [157]. GLP-1 signaling was associated with multiple beneficial effects in heart failure pathophysiology through systemic and cardiac actions. The systemic effects are related to the mediation of insulin secretion and sensitivity and subsequent glucose and lipid metabolism improvement in the pancreas, liver, and adipose tissue. GLP-1 has direct cytoprotective effects in cardiac cells and atheroprotective effects in vascular cells [158]. 

Incretin-based therapies have been proved to interact with renin–angiotensin–aldosterone system (RAAS) both in animal and clinical studies, therefore suggesting a connection between classical heart failure pathways and incretins. The mechanisms of RAAS components being influenced by incretins are Angiotensin (Ang) II inhibition, Na+/H+ exchanger isotope 3 (NHE3) modulation, regulation of central nervous system (CNS)-induced RAAS activation, and decrease in AT1/AT2 ratio. GLP-1 is involved in Ang II inhibition through PKA-dependent pathways, direct effect on renal juxtaglomerular cells, inhibition of proximal sodium transport, subsequent modulation of tubule-glomerular feedback, and direct Ang II down-regulation [159]. In a study based on both in vivo and in vitro experiments, GIP infusion suppressed cardiomyocyte hypertrophy and apoptosis and interstitial fibrosis in the ventricular wall induced by Ang II via GIP/GIPR/cAMP/phosphorylated Akt axis. According to these morphological changes, GIP infusion produced the downregulation of TGF-β1 and HIF-1α and inhibited the mRNA expression of B-type natriuretic peptide and TGF-β1, which were upregulated by Ang II in vitro [160]. Liraglutide, a GLP-1 analog, significantly reduced cardiac hypertrophy and fibrosis and improved cardiac function in a non-diabetic animal model of cardiac hypertrophy induced by Ang II or pressure overload by modulation of PI3K/Akt1 and AMPKα signaling [161]. 

Liraglutide proved to have more beneficial effects in animal studies of experimentally induced heart failure. In cultured cardiomyocytes exposed to IL-1β, liraglutide ameliorated the ROS production, mitochondrial function, and triglyceride metabolism with subsequent lipid accumulation via regulation of acetyl-CoA carboxylase (ACC) in an AMPK-dependent manner, suggesting a therapeutic implication for GLP-1 agonists in cardiac inflammation [162]. A contradictory observation was made in an animal model of spontaneous dilated cardiomyopathy—Liraglutide deteriorated cardiac function and produced increased fibrosis and cardiac enlargement, effects associated with an increased consumption of carbohydrates and energy caused by GLP-1 agonist administration. The appropriate energy supply with glucose in rats treated with Liraglutide ameliorated the cardiac function and metabolism, suggesting that, in heart failure patients with incretins-based therapy, the intake of carbohydrates should be carefully considered [163]. However, in studies based on human subjects, treatment with GLP-1 agonists proved to have beneficial effects. A meta-analysis of eight randomized controlled trials proved that GLP-1R agonists, regardless of molecular structure, reduced the risk of major acute cardiovascular events, all-cause mortality, hospitalizations for heart failure, and worsening kidney function in patients with type 2 diabetes; thus, GLP-1R-based therapies represent an important method to reduce the mortality and morbidity in diabetic patients [164]. In a small group of patients with severe chronic heart failure, chronic GLP-1 infusion improved left ventricular function, functional status, and quality of life, benefits observed both in diabetic and nondiabetic patients [165]. 

A DPP-4 inhibitor, alogliptin, prevented the contractile dysfunction, ventricular remodeling, and cardiomyocytes apoptosis induced by pressure overload in an animal model of ventricular dysfunction via GLP-1R stimulation of cAMP/PKA/EPAC1 signaling pathway that enhance pro-survival proteins [166]. However, the use of some DPP-4 inhibitors increased the risk of heart failure and worsened the evolution of heart failure in diabetic patients. This is explained by the activation of the sympathetic nervous system and stimulation of β-adrenergic receptors through interference of DPP-4 inhibitors with the degradation of substance P, stromal cell-derived factor 1, and neuropeptide Y, causing cardiac cell apoptosis, through a Ca++/calmodulin-dependent protein kinase II pathway [167]. 

Arterial hypertension is a common cause of heart failure, particularly for HF with preserved EF. In a spontaneously hypertensive, heart failure-prone (SHHF) animal model, GLP-1 infusion for 3 months resulted in improved survival, reduced cardiomyocyte apoptosis via downregulation of caspase-3, preserved left ventricular function and left ventricular mass index and improved myocardial glucose uptake [168]. GLP-1 intestinal secretion was increased as an adaptive response in a hypertensive heart failure experimental model. Miglitol, an α-glucosidase inhibitor, stimulated GLP-1 production, which improved cardiac dysfunction and prevented cardiac remodeling via GLP-1R/PKA. Miglitol, together with enhanced GLP-1 production, mediated the mitochondrial fusion and function by PKA activation and release of mitochondrial fusion-related proteins, leading to increased ATP content, suggesting that GLP-1 can ameliorate cardiac dysfunction by acting at the mitochondrial level [169]. 

The effects of incretins in experimental myocardial ischemia as a precursor of heart failure development were evaluated in both animal and clinical studies. In both in vivo and ex vivo models of experimental myocardial ischemic injury, GLP-1(28–36), a GLP-1 metabolite generated by neutral endopeptidase, proved to exert cardioprotective effects regarding ischemic cardiac dysfunction, infarct size, and coronary vascular cells. The mechanisms involved were the activation of soluble adenylyl cyclase (sAC) and increased cAMP/PKA activity in coronary smooth muscle cells and endothelial cells, with the consecutive phosphorylation of endothelial nitric oxide synthase (eNOS). An increased intracellular ATP level was observed due to the GLP-1(28–36) mediation of mitochondrial trifunctional protein-α (MTPα) causing a change in the basal metabolism, from fatty acid oxidation to more efficient glucose oxidation and oxygen-sparing glycolysis and further higher levels of cAMP and ATP, reducing the metabolic oxidative stress [170]. On the contrary, in an animal model of myocardial infarction, selective GIPR inactivation reduced the ventricular injury, improved survival, and increased myocardial triacylglycerol (TAG) content by decreasing hormone-sensitive lipase (HSL) phosphorylation via PKG/ERK pathway, suggesting an adaptive role for GIPR signaling in ischemic conditions [171]. 

Exenatide, a GLP-1 analog, reduced MI size and prevented MI-induced myocardial remodeling and contractile dysfunction in a porcine model of left circumflex artery ligation and subsequent reperfusion. Exenatide treatment increased the expression of phosphorylated Akt, antiapoptotic protein Bcl-2, and the activity of antioxidant enzymes, such as superoxide dismutase and catalase, and decreased caspase-3 activity, suggesting that GLP-1 signaling mediates apoptosis and oxidative stress following ischemia/reperfusion injury [172]. Treatment with sitagliptin, a DPP-4 inhibitor, in ischemic normoglycemic rats attenuates the high levels of resistin associated with MI via GIP-dependent pathways including Akt/PI3K signaling. GIP infusion decreased resistin levels, an adipokine involved in inflammation and atherosclerosis in a similar way to sitagliptin, suggesting that DPP-4 inhibition produced by sitagliptin provides beneficial effects through the mediation of GIP. Moreover, sitagliptin showed antiarrhythmic effects by mediating sympathetic innervation via the PI3K pathway and nerve growth factor (NGF) expression [173]. 

Pre-treatment with GLP-1(7–36)amide in patients with ischemic heart disease caused by balloon occlusion protected against LV systolic and diastolic dysfunction and improved the recovery of LV performance during reperfusion, without detectable changes in cardiac glucose metabolism, suggesting an independent mechanism for GLP-1 not related to glucose metabolic effects [174]. Intravenous infusion of GLP-1(7–36)amide improved global and regional function of the left ventricle, assessed by velocity, strain, and strain rate, and improved the post-ischemic myocardial stunning, particularly in ischemic segments, in patients with coronary artery disease and ischemic dysfunction induced by dobutamine stress echocardiography [175]. 

In diabetic patients, the accumulation of methylglyoxal (MG), a precursor of advanced glycation end products (AGEs) and a source for the synthesis of intracellular AGEs, has been related to the development of diabetic cardiomyopathy. In rat cardio-myoblast cells, MG produced oxidative stress activation, myocyte injury, apoptosis, and mitochondrial dysfunction. GLP-1R stimulation with exendin-4 inhibited these effects through activation of the cAMP/EPAC/PI3K/Akt signaling pathway. Exendin-4 via GLP-1R improved mitochondrial membrane potential and the expression of genes involved in mitochondrial function, suggesting a therapeutical role for GLP-1R stimulation in mitochondrial function and oxidative stress [176]. Calcium disturbances are frequently involved in heart failure pathophysiology. Treatment with exendin-4, as a GLP-1R agonist in a MI animal model, decreased the size of the infarcted myocardium, prevented the dilation of cardiac chambers and the progressive remodeling, improved the systolic function of the heart and suppressed myocyte hypertrophy and fibrosis, effects mediated by the circulating GLP-1 and ventricular GLP-1R. At the molecular level, exendin-4 activated the eNOS/cGMP/PKG pathway and inhibited the Ca2+/calmodulin-dependent kinase II (CaMKII) pathway, improving calcium homeostasis via modulation of the expression of proteins responsible for calcium handling: sarcoplasmic reticulum Ca2+uptake ATPase (SERCA2a), phosphorylated phospholamban (PLB), Cav1.2 and phosphorylated ryanodine receptor (RyR) [177]. Exendine-4 decreased inflammation and interstitial fibrosis in the myocardium following MI, besides the protective effects on cardiac function, chamber size, and cardiomyocyte survival. These effects were associated with the downregulation of gene expression of extracellular matrix remodeling markers and pro-inflammatory cytokines and the modulation of Akt/glycogen synthase kinase 3b (GSK-3b) and Smad 2/3 signaling, pathways involved in the fibroblast-driven remodeling and extracellular matrix turnover [178]. GLP-1(9–36)amide, the inactive form of GLP-1, has been involved in protection against ventricular remodeling following experimentally-induced acute myocardial infarction in mice, improving diastolic parameters via the mediation of macrophages infiltration and extracellular matrix composition [179].

Heart failure with preserved ejection fraction (HFpEF), besides being frequently encountered and diagnosed nowadays, does not have an optimal therapeutical approach. In a rat model of HFpEF, GLP-1 infusion improved survival, ameliorated the parameters of diastolic dysfunction, and reduced left ventricular stiffness and pulmonary congestion. GLP-1 infusion was associated with a shift of cardiac metabolism towards glucose oxidation, suggesting a favorable metabolic mechanism of GLP-1 in diastolic dysfunction [180]. In western-diet-fed mice and diastolic dysfunction, DPP-4 inhibition improved diastolic function and insulin resistance, reduced myocardial oxidative stress and fibrosis, and modulated the endothelial vascular ultrastructure [181], indicating that incretins may provide new therapeutic options for patients with HFpEF. 

The main experimental studies conducted on animals and cultured cell lines and the following mechanisms of incretins are exposed in Table 2.

- Ghrelin

The beneficial effects of ghrelin administration observed in heart failure studies both on animal and human subjects suggested that ghrelin may play a role as a therapeutic agent in heart failure. The cardioprotective actions regarding cardiac hypertrophy, fibrosis, calcium handling, and cardiac remodeling following ischemic injury demonstrate that ghrelin acts on different pathways in the pathophysiology of both heart failure with preserved and reduced ejection fraction [182]. The molecular mechanisms involved are modulation of autophagy, ionotropic actions, anti-apoptotic and anti-inflammatory pathways activation, and regulation of the autonomic nervous system [183].

In chronic heart failure patients, low ghrelin levels were a marker of increased severity and worse prognosis. Ghrelin levels were independently associated with adverse cardiac events in a multivariate analysis, thus suggesting that ghrelin is a possible new guiding marker in heart failure management [184]. In cachectic patients with chronic heart failure, plasmatic levels of ghrelin were elevated and were positively correlated with serum levels of GH and TNF-α, suggesting a compensatory mechanism activated in conditions of accentuated catabolism in chronic heart failure [185]. Furthermore, patients with dilated cardiomyopathy had lower levels of ghrelin, both acylated and un-acylated, than control subjects, and ghrelin levels were inversely associated with the duration and the left ventricular ejection fraction, proving a complex relationship between ghrelin and heart failure development [186]. These contradictory results may be explained by the differences between the subject with different heart failure stages and diverse causes of heart failure. In advanced stages of heart failure, when the ejection fraction is markedly decreased, the ghrelin/GHS-R axis is altered: ghrelin secretion is impaired and GHS-R1a expression is compensatorily increased [187]. Tissue samples from patients with valvular heart disease with or without coronary artery disease and without reduced LVEF provided evidence for a positive correlation between ghrelin and GHS-R levels in the affected areas and between ghrelin, GSH-R, and BNP and the contractility marker, SERCA2, in the left ventricle. The authors also detected a positive correlation between ghrelin and BNP levels and showed that these two peptides are colocalized in the myocardium, suggesting that in valvular heart disease, in the subclinical stage, the deficiency of endocrine signaling may be an important key factor that drives myocardial dysfunction. The correlation with the contractility marker could imply an adaptive cardioprotective mechanism before decreased EF [188]. The infusion of acyl and des-acyl ghrelin in animals with pacing-induced HF improved the cardiac metabolism and energy balance, leading to enhanced free fatty acid oxidation and reduced glucose oxidation, suggesting that impaired ghrelin’s secretion in advanced stages of heart failure may be responsible for metabolic alterations [189]. In chronic heart failure patients, ghrelin administration increased the ejection fraction of the LV, decreased the LVESV, and improved exercise capacity and muscle wasting [190].

Ghrelin showed cardioprotective actions in the ventricular remodeling after myocardial infarction (MI): improvement in cardiac contractility, enhancement of the antioxidant activity of the myocardium, decrease in inflammation, fibrosis, and apoptosis via multiple signaling axis: activation of Raf-1-MEK1/2-ERK1/2 and consecutive inactivation of pro-apoptotic proteins [191] and/or activation of JAK2/STAT3 and inhibition of STAT1 pathway [192]. After MI, the ventricular remodeling is associated with myocardium fibrosis and ECM turnover, involved in contractility dysfunction, and carries the risk of life-threatening arrhythmias. Ghrelin has proved to exert inhibitory effects on fibrosis after MI via the downregulation of activin A (Act A), a member of the TGF-β superfamily and a pro-fibrotic agent, and consequent adjustment of the imbalance between Act A and its blocker-follistatin (FS) [193], inhibition of endothelial-to-mesenchymal transition, an essential process in myocardium fibrosis initiation, in a GHSR-1a/AMPK/Smad7-dependent manner, disrupting TGF-β1 signaling [194], and modulation of oxidative stress reaction by activation of nuclear factor-erythroid 2-related factor 2 (Nrf2) and inhibition of NADPH/ROS pathway [195]. Ghrelin manifested anti-apoptotic effects at the ultra-structural level following MI: preservation of the mitochondrial structure, amelioration of microfilament appearance and organization, increase in the number of endoplasmic reticulum intracellular organelles, and improvement in nucleus structure [196]. 

After an ischemic injury, cardiac fibrosis and ventricular remodeling exhibit a pro-arrhythmic risk. Ghrelin reduces this risk and improves survival and cardiac function by the preservation of the electrophysiological properties of the cardiomyocytes, regulating the L-type Ca channels and sodium channels, and maintaining the action potential normal amplitude and duration. Moreover, ghrelin inhibited cardiac cell apoptosis in a GHS-R1a/MAPK-dependent manner [197], prevented the loss of connexin-43 within the left ventricle and modulated cardiac autonomic innervation [198] with suppressing effects over cardiac sympathetic nerve activity via vagal afferent fibers [199,200]. 

Myocardial ischemia/reperfusion (IR) injury provides deleterious effects following reperfusion strategies in acute coronary syndromes. Ghrelin has been involved in the improvement in cardiac function following ischemia/reperfusion by decreasing the infarction area, apoptosis, and inflammatory reaction and ameliorating the oxidative stress via modulation of Toll-like receptors 4 (TLR4)/NLRP3 inflammasome signaling pathways. In cultured cardiomyocytes, ghrelin inhibited the LPS-mediated stimulation of NLRP3, caspase-1, and IL-1β, showing anti-inflammatory properties [201]. The inhibition of pro-inflammatory and apoptosis key players was an effect of ghrelin administration in multiple animal studies of myocardial ischemia/reperfusion injury through activation of different pathways, including inflammatory axes, such as High mobility group box 1 (HMGB1)/TLR4/NF-κB pathway [202,203]. Ghrelin preserved cardiac contractility following IR injury and produced a positive inotropic effect by the maintenance of sarcoplasmic reticulum calcium content and intracellular calcium homeostasis [98]. In a model of myocardial ischemia/reperfusion injury, a bioactive fragment of un-acylated ghrelin-UAG _6–13_ showed cardioprotective effects by the decrease in the infarcted area and inflammation markers and improvement in myocardial hemodynamic properties, the effects independent of the GHS-R1a activation [204]. 

The upregulation of Ang II is a well-known mediated pathway in the development of heart failure. Ghrelin inhibited Ang II-induced myocardial hypertrophy and fibrosis by upregulating PPAR-γ and inhibiting the expression of TGF-β1 and associated downstream proteins [205]. Another mechanism involved in Ang II deleterious effect on heart failure is cardiomyocyte apoptosis. Ghrelin administration reduced the Ang II induced-apoptosis of cultured cells and favored the expression of miR-208, a miRNA family responsible for the modulation of several apoptosis pathways, including caspase, with protective effects against Ang II [206]. The antiapoptotic effect of ghrelin can also be explained by the downregulation of miR-122 and the subsequent overexpression of Sestrin-2, both important regulators of cardiac cell apoptosis [207]. 

Hypertrophy of the myocardium is an adaptive reaction during heart failure development, but it has negative effects in the long term, with the risk of sudden death. Ghrelin showed beneficial effects in attenuating cardiac hypertrophy and consequent fibrosis, inflammation, and apoptosis and improving autophagy activity via the Ca2+/Calmodulin-dependent protein kinase (CaMKK)/AMPK pathway [208]. The activation of the cholinergic anti-inflammatory pathway was a different mechanism activated by ghrelin that ameliorated cardiac hypertrophy [209]. 

Metabolic-induced cardiomyopathy is related to numerous cardiovascular risk factor and metabolic dysregulation. Ghrelin protected against obesity-induced cardiomyocytes apoptosis and the pro-inflammatory environment caused by the high-fat diet or palmitic acid by the regulation of lncRNA H19/miR-29/IGF-1 [210] and Homeobox transcript antisense RNA (HOTAIR)/miR-196b/IGF-1 axis [56], novel signaling cascades involved in metabolic cardiac injury. In a type 2 diabetic-induced cardiomyopathy mouse model, infusion with des-acyl ghrelin improved contractile dysfunction, reduced cardiac fibrosis, and enhanced the autophagic signaling via modulation of pro-survival cellular AMPK/ERK1/2 signaling pathways, suggesting a cardioprotective role of des-acyl ghrelin [211]. 

Doxorubicin, an anthracycline tumor suppressing drug, activates multiple pathways that lead to cardiotoxicity. In doxorubicin-induced cardiomyopathy, ghrelin supplementation improved the cardiac dysfunction, cell viability, survival, suppressed apoptosis, and excessive autophagy induced by doxorubicin through inhibition of oxidative stress, activation of mTOR phosphorylation via AMPK suppression and p38-MAPK stimulation [212].

- CCK

In clinical studies of heart failure patients, CCK levels predicted mortality in elderly women, suggesting an important role of CCK in cardiovascular risk assessment [213]. In an animal model of myocardial infarction, the upregulation of CCK was correlated with markers of heart failure progression, such as BNP levels, left ventricular end-systolic diameter, ejection fraction, and shortening fraction [214]. However, CCK-8 administration, a sulfated carboxyterminal octapeptide and a major bioactive segment of CCK improved the ventricular function and attenuated myocardial fibrosis and ventricular remodeling in an animal study of myocardial infarction [215]. CCK-8 has been studied as a protective factor against Ang II-induced apoptosis via the CCK-1 receptor and PI3K/Akt signaling pathway [216].

#### 3.2.2. Gut Microbiota-Derived Products

- TMAO

TMAO, as a marker of gut dysbiosis, is involved in many steps of heart failure development and carries the potential of being both a predictor and a therapeutic target [217]. TMAO levels, increased in patients with chronic heart failure, were an independent predictor of cardiovascular mortality in the long-term follow-up [218] and were associated with NYHA functional class and ischemic etiology of heart failure [219]. Moreover, serum trimethyl lysine (TML) levels, a TMAO precursor, were associated with the presence and severity of heart failure, suggesting a higher risk for cardiovascular death, re-hospitalization, and all-cause mortality in heart failure patients [220]. In the Biology Study to Tailored Treatment in Chronic Heart Failure (BIOSTAT-CHF) system, TMAO levels were correlated with mortality and/or rehospitalization, and contrary to BNP levels, TMAO levels have not been reduced by the guideline-recommended pharmacological treatment, noticing that further therapeutic measures need to be considered in heart failure treatment [221]. TMAO serum concentration, increased in symptomatic heart failure patients, did not lower after heart transplant or LVAD implantation and was independent of makers of inflammation, oxidative stress, endotoxemia, or gut microbiota composition after multivariable adjustment. After LVAD implantation or heart transplant, the inflammation biomarkers were reduced, but the oxidative stress and endotoxemia markers were increased, suggesting additional mechanisms to hemodynamic improvement, such as the relationship between gut microbiota and inflammation, to be responsible for the heart failure-associated complications [222]. In patients presenting with heart failure with preserved ejection fraction, in the Developing Imaging and plasma biomarkers in describing Heart Failure with Preserved Ejection Fraction (DIAMONDHFpEF) cohort, TMAO levels, increased in patients with left ventricular filling pressure along with BNP levels, were useful for better risk stratification for long-term mortality, particularly in patients with low levels of BNP [223]. 

In heart failure, the dysfunctional gut–blood barrier induced by reduced intestinal blood flow, decreased thickness of colonic mucosa, and alterations of the tight junctions’ proteins allow an increased passage of the TMAO precursor, TMA, into the blood and consecutive higher plasmatic levels of TMAO that could explain the observed increased TMAO levels in heart failure patients [224]. However, TMAO can activate pathophysiological cascades involved in heart failure development. In an experimental study of pressure overload-induced heart failure, choline or TMAO administration exacerbated the left ventricular dysfunction, cardiac enlargement, and pulmonary edema, increased the levels of BNP, and exacerbated interstitial and perivascular ventricular fibrosis [225]. TMAO was demonstrated to induce cardiac fibrosis and hypertrophy in a TGF-β1/Smad3-dependent manner [226]. TMAO stimulated cardiac inflammation and fibrosis responsible for the systolic and diastolic dysfunction noticed in mice fed with a Western diet, adding supplementary mechanisms for the hypothesis of the gut microbiota as a part of the development of heart failure [227]. Moreover, TMAO administration showed negative effects regarding cardiac contractility and intracellular calcium handling and reduced the sarcomere fraction of shortening and the maximal rate of shortening and re-lengthening, and prolonged the time needed for the removal of cytosolic calcium. Transmission Electron Microscopy (TEM) images showed that cardiomyocytes exposed to TMAO contained increased glycogen accumulation, a higher number of mitochondria, and paranuclear lipofuscin-like droplets, suggesting an impaired energetic metabolism and protein oxidative damage [228]. 

However, some studies showed the protective effects of TMAO regarding heart failure development. In male spontaneously hypertensive rats, TMAO administration in low doses improved cardiac fibrosis, NT-pro BNP levels, and left ventricular end-diastolic pressure, suggesting the beneficial effects of TMAO in pressure overload diastolic dysfunction [229]. In a rat model of right ventricular heart failure, long-term TMAO administration preserved the right ventricular function via improvement in the mitochondrial energy metabolism through fatty acid oxidation and decrease in pyruvate metabolism, showing preconditioning-like metabolic and cardioprotective effects during heart failure initiation [230]. However, in another study, TMAO impaired mitochondrial metabolism by alterations of fatty acid oxidation and pyruvate metabolism, resulting in cardiac energetic disturbances, suggesting that TMAO could have a dual role in cardiac pathophysiology depending on specific conditions [231].

- LPS

LPS, as a marker of endotoxemia, has been associated with cardiac dysfunction and heart failure development. In patients with chronic heart failure, LPS responder status, related to the potential to secrete TNF-α to increasing doses of LPS, was an independent predictor of mortality after multivariable adjustment [232]. Moreover, in decompensated chronic heart failure, the gut function of active-carrier-mediated transport was reduced due to gut edema. The high serum LPS levels were associated with an increased level of cytokines, suggesting a complex relationship between decompensated heart failure, edema in the gut wall, gut barrier dysfunction, and epithelial disturbances [233]. 

The inflammatory reaction that drives heart failure progression may be exacerbated by LPS. LPS increased the expression of syndecan-4 via NF-κB, a member of the syndecan superfamily involved in tissue inflammation and wound healing, and the release of cytokines, adhesion molecules, and extracellular matrix remodeling markers to promote immune cell recruitment, immunity activation, and cardiac remodeling [234]. Additional pro-inflammatory pathways influenced by LPS are the upregulation of miR-203 that inhibits the expression of nuclear factor interleukin-3 (NFIL3) and activates the production of cytokines and pro-apoptotic factors [235], overexpression of long non-coding RNA SRY-Box Transcription Factor 2 (SOX2) overlapping transcript (SOX2OT) and subsequent downregulation of miR-215-5p and release of ICAM-1, as a pro-inflammatory, pro-apoptotic and adhesion molecule [236]. Overexpression of Na^+^/Ca^2+^ exchanger 1 (NCX1) induced by LPS promoted cardiac hypertrophy [237]. LPS was involved in cardiomyocyte contractile dysfunction and cardiac calcium handling dysregulation through the decrease in the L-type calcium channels function and oxidative modification of sarcoendoplasmic reticulum Calcium-ATPase (SERCA)- Cys^674^ sulphonylation [238]. 

Activation of TLR4 by LPS produced cardiac inflammation, oxidative stress activation, mitochondrial dysfunction, cardiac fibrosis, and cellular apoptosis via SIRT2 inhibition and p53 acetylation [239]. In a rat model of myocardial infarction, the authors demonstrated that after myocardial injury, TLR4 expression is upregulated and the binding capacity of LPS is increased, promoting cardiac inflammation and exacerbation of heart failure [240]. LPS induces cardiac fibrosis through many mechanisms that comprise the upregulation of cardiac fibrosis mediators and enhancement of cellular migration in cardiac fibroblasts via the ERK1/2 signaling pathway [241] and the activation of NADPH oxidase 2 (NOX2) and the downregulation of miR-29 [242].

### 3.3. Atrial Fibrillation

Atrial fibrillation is the most common arrhythmia worldwide and is part of the cardiometabolic spectrum of diseases, more frequently encountered in patients with a high cardiovascular risk. The traditional risk factors for atrial fibrillation are coronary artery disease, hypertension, diabetes, heart failure, and obesity, all of them characterized by the description of the gut dysbiosis hypothesis in pathophysiological processes. Therefore, the involvement of gut metabolites in the development and progression of atrial fibrillation has been reviewed. Gut microbiota metabolites, such as TMAO, LPS, secondary bile acids, and indoxyl sulfate have been associated with the remodeling of the autonomic activity, calcium handling mechanisms, the atrial electrical properties and the atrial structure, increasing the risk for atrial fibrillation causing re-entrant substrate and triggered activity [243]. 

TMAO was associated with the development and progression of atrial fibrillation, independent of other AF traditional risk factors [244]; the enzymes involved in the synthesis of TMA, as a precursor of TMAO, the bacteria genera correlated with these enzymes [245], and the metabolites from choline biochemical cascade, such as choline, betaine, and dimethylglycine [246] were described as part of the association. In an experimental model of AF, TMAO produced atrial electric instability, increased AF inducibility, and aggravated the acute electrical remodeling by enhancement of the cardiac autonomic activity and remodeling and activation of the pro-inflammatory p65 NF-κB signaling pathway, showing a mechanism that may play an important role in the perpetuation of atrial fibrillation [247]. 

In patients with nonvalvular atrial fibrillation from the ATHERO-AF cohort, circulation levels of LPS were correlated with PCSK9 levels, and the concomitant increased levels were associated with a higher risk of cardiovascular events. Possible mechanisms for this finding were the activation of NADPH oxidase and a prothrombotic effect of LPS produced by the increase in PCSK9 [248]. In the same cohort of patients, LPS levels were correlated with a reduced level of antioxidant enzymes, indicating that LPS could promote impaired antioxidant activity. Patients with higher levels of LPS and reduced antioxidant enzyme glutathione peroxidase 3 had a higher risk of cardiovascular events [249]. LPS and collagen type-1 C-terminal telopeptide (CITP) independently predicted the risk for AF recurrence after radiofrequency ablation. LPS may precipitate atrial fibrillation recurrence through systemic inflammation pathways [250]. 

In an animal model of age-related atrial fibrillation, high LPS levels, as a marker of a dysfunctional gut barrier, and increased glucose levels produced by impaired glucose tolerance activated the NLRP-3 inflammasome and produced the development of atrial fibrillation via atrial remodeling and fibrosis. The aging process changed the gut microbiota composition and affected the gut barrier integrity, leading to increased gut permeability of LPS into the blood [251]. Moreover, LPS, via stimulation of pro-inflammatory macrophages, increased the incidence of atrial fibrillation, produced atrial electrical remodeling, and reduced the atrial effective refractory period and L-type calcium currents. The interaction between LPS and atrial myocytes was mediated by the secretion of IL-1β by macrophages, which inhibited the expression of atrial myocyte quaking protein (QKI) and a1C subunit of L-type calcium channel (CACNA1C) [252]. LPS reduced the effective refractory period, widened the window of vulnerability of atrial appendage cells, promoted the expression and lateral distribution of connexin 43, and activated NF-κB signaling via a1-adrenergic receptor (α1-AR) in an animal model of atrial fibrillation, indicating that chronic inflammation represented by LPS may play a role in the atrial fibrillation pathogenesis [253].

## 4. Gut Molecules and Metabolic Disorders: Focus on the Diabetes Mellitus

### Gut Hormones

- Incretins

Regarding the potential effect of incretins in the pathophysiology of diabetes mellitus (DM), both GIP and GLP-1 bind to their specific receptor on the pancreatic cells, GIPR and GLP-1R, which are part of the family of G protein-coupled receptors [254,255]. They exert agonist effects on the pancreatic function such as promoting glucose-dependent insulin secretion, decreasing beta-cell apoptosis, and promoting beta-cells proliferation, as described above, but also antagonist action on glucagon secretion, with the stimulatory effect of GIP and the inhibitory effect of GLP-1 [256]. Due to the beneficial effect on glycemic control, several GLP-1 analogs and GLP-1 receptor agonists (GLP-1RA), resistant to the DPP-4 action, were developed and received approval for use in the treatment of T2DM. The first approved drug with an incretin-like mechanism of action in the pharmacotherapy of T2DM was exenatide (the synthetic version of exendin-4—a 39 amino acid-long peptide, isolated from the venom of the Gila monster lizard Heloderma suspectum) under the commercial name of Byetta—U.S. Food and Drug Administration (FDA) approval in 2005 [257] (sitagliptin, first DPP-4 inhibitor, was approved by FDA in October 2006). There was a long way from two daily injections (Byetta—exenatide) to oral administration of a GLP-1 RA (Rybelsus—oral semaglutide) and incretin therapy, which, due to its pleiotropic effect, is a very attractive therapeutic area, with many molecules under development. Recently, a dual GIP/GLP-RA also received approval for use in the treatment of T2DM [258]. In Table 3, we attempted to summarize the GLP-1 analogs, GLP-1RA, and dual GIP/GLP-RA available in 2022 for use in the treatment of T2DM. 

Beyond glycemic control, GLP-1RAs exert beneficial effects on the neurological system (Alzheimer’s disease, Parkinson’s disease, stroke, chronic pain), cardiovascular system (atherosclerosis, heart failure), endocrine system (polycystic ovary syndrome), and influence the control of obesity, fatty liver disease and inhibitory effect on the growth of several types of tumors (ovarian, breast, prostate, and pancreatic), but further research is ongoing or needed to clarify the complete picture of pleiotropic effects of GLP-1Ras [25]. In addition, other dual agonists (GLP-1RA and glucagon receptor agonists, such as oxyntomodulin) or triple agonists (GIP/GLP-1/glucagon receptor agonists, such as YAG-glucagon) are in development for the treatment of T2DM [268].

- Ghrelin

Recent findings showed that GHR is much more than a hunger hormone, exhibiting pleiotropic effects, among which there are regulation of glucose homeostasis by inhibiting insulin secretion (resulting in increased circulating glucose levels) and modulating gluconeogenesis and glycogenolysis [268]. 

Dezaki et al. showed that endogenous GHR in pancreatic islets of rats restricts insulin release by attenuating Ca^2+^ signaling in the beta-cells [269]. On the other hand, GHR inhibits cell apoptosis of the beta-cells induced by lipotoxicity, transforming GHR agonists into a promising therapeutic path to preserve and improve the function of the remaining beta-cells in the early stages of both T1DM and T2DM. In an interesting study, Irako et al. showed that GHR promotes the regeneration of beta-cells in diabetes-prone animals (streptozocin-treated newborn rats), suggesting that early administration of GHR may help prevent the development of diabetes after beta-cell destruction [270].

In newly diagnosed T1DM children, it was observed that acylated ghrelin levels were lower at diagnosis, with a significant increase at 1 month and normalizing at 4 months, suggesting that the body mass index is not the main predictor of ghrelin and it may play an important role in the metabolic adaptation in this disease [271]. In mice with streptozotocin-induced diabetes, increased plasma ghrelin levels, hyperphagia, and altered gastric emptying were observed. Verhulst et al. showed that ghrelin is involved in diabetic hyperphagia by central mechanisms (affecting the expression of NPY and AgRP in the hypothalamus) and the acceleration of gastric emptying is not the cause of diabetic hyperphagia [271].

Few antagonists of the receptor of GHR and GOAT antagonists are currently under investigation in clinical studies [272]. A single dose administration of YIL-870 (GHS-R1a antagonist) improved glucose homeostasis in a glucose tolerance test in the rat (by promoting glucose-dependent insulin secretion). Daily oral administration of the antagonist to obese mice (diet-induced) reduced food intake and weight loss (up to 15%) by selective fat mass loss [273]. Several GOAT antagonists are in clinical studies as potential treatments for a range of diseases, including diabetes. The finding that GHR is the only substrate for GOAT—“one enzyme—one substrate”—suggests the potential to develop enzyme inhibitors with minimal potential for unexpected and undesirable off-target pharmacodynamic effects [274].

- PYY

PYY_3–36_ has an important role in appetite suppression due to its effect on hypothalamic neurons that express Y2 receptors, with a consecutive role in weight regulation [275]. The link between obesity and T2DM is well known. Due to the observed effect of reducing food intake, PYY has the potential to be a useful tool for the fight against obesity and T2DM. In a human study, with 28 non-obese healthy male participants, Degen et al. observed that the intravenous infusion of PYY_3–36_ had a dose-dependent inhibitory effect on food, caloric and liquid consumption, with maximal inhibition of 35%, 32%, and 18%, respectively. The significant p values were observed at pharmacological plasma concentrations (0.4 and 0.8 pmol/kg/min), but not at physiological plasma concentrations (0.2 pmol/kg/min) [276]. The effects were explained by the direct central action on satiety, gastro-intestinal effects (slowing of the gastric motility), and the modulation of ghrelin concentration (i.v. PYY infusion decreased pre- and postprandial levels of ghrelin), although the precise mechanism was not elucidated. 

Human NPY receptors subtype Y1 (hY1) are present in numerous tissues such as the brain, aorta smooth muscle, kidney, testis, placenta, and pancreas [277]. All three members of the NPY family (NPY, PYY, and PP) can link to the hY1 receptor from the surface of the beta-cells, which is coupled with a G-complex protein. After receptor–substrate binding, intra-cellular cascades mediate the transcriptional process, modulating the function of genes involved in the development, maturation, and function of the cells. The activation of the hY1 receptor harms insulin secretion from beta-cells, and it was observed in mice that by transplanting islets with a deficiency of Y1 receptors, normoglycemia can be faster restored in alloxan-induced diabetes. In the same study, Loh et al. showed that BIBO3304, a highly potent and selective Y1 receptor antagonist, enhances human islet function and transplant outcome by promoting insulin secretion and enhancing β-cell proliferation [277]. PYY has an inhibitory effect on insulin secretion via Y1 receptors and those receptors are negative regulators of insulin release from the pancreatic beta-cells under a physiological glucose load, suggesting a potential role of PYY in beta-cells rest. Ramracheya et al. demonstrated that bariatric surgery (Roux-en-Y gastric bypass) normalizes glycemia in diabetes by restoring insulin and glucagon secretion in a PYY-independent manner [278].

- Oxyntomodulin

Oxyntomodulin (OXM) is a 37-amino acid peptide gut hormone secreted by L cells of the intestine, as a response to food intake and in correlation with the caloric load. The circulating levels of OXM increase with age, whereas no change was noticed according to sex [157]. It is inactivated by the DPP-4 enzyme (the half-life of OXM in plasma is 12 +/− 1 min) [279] due to the removal of the first two N-terminal amino acids of the peptide. Its biological effects are attributed to a dual activation of GLP-1R and glucagon receptors (Gcg-R). 

It was first observed in mice that co-administration of GLP-1 and Gcg (in doses that individually did not significantly affect feeding) have an anorectic effect and produced neuronal activation in the area postrema and central nucleus of the amygdala, decreasing food intake and increasing energy expenditure. Co-administration of GLP-1 and Gcg prevented the acute hyperglycemia induced by Gcg alone [280]. Later, Cohen et al. observed in a study that enrolled 13 normal-weight, healthy subjects, that OXM infusion significantly decreased the ad libitum energy intake by 19.3 +/− 5.6%, with an inhibitory effect lasting up to 12 h (11% reduction in food intake), without nausea or interference with food palatability. They also observed that the pre-prandial level of ghrelin was significantly suppressed by OXM (by 44 +/− 10%) [281]. Chronic subcutaneous administration of OXM (4 weeks, 3 times/day, 30 min before each meal) produced significant weight loss of 2.3 +/− 0.4 kg versus 0.5 +/− 0.5 kg in the control group in a randomized, double-blind, parallel-group study with healthy overweight and obese volunteers [157].

OXM has an affinity of 1/50–1/100 for GcgR and 1/100 for GLP-1R. The overall effect of oxyntomodulin on glucose metabolism is less clear since the actions on hepatic glucose production and insulin secretion would be expected to pull in different directions due to this co-stimulation [282]. Recently, Benson et al. reported that an acylated peptide analog of oxyntomodulin administered for 12 or 16 weeks in T2DM patients induced HbA1c reduction from baseline ranged from −1.56% to −2.16% in OXM-treated group versus −0.43% to −0.70% in control subjects, and mean body weight changes from baseline ranged from −2.30 kg to −11.24 kg in the treated group versus −0.35 kg to −2.03 kg in control subjects [283]. 

## 5. Conclusions

The use of incretin-based therapies in diabetes mellitus initiated the idea of a potentially beneficial role of incretins in the prevention of atherosclerosis and heart failure, mediating different signaling pathways in cardiac and vascular cells. Although some controversial results were observed in studies using incretin infusion, incretin-based therapies have to be considered in the treatment of diabetic patients with associated cardiovascular diseases. Other gut hormones, particularly ghrelin, have additional beneficial properties, both in cardiac disorders and diabetes mellitus. Gut microbiota-derived products are important key factors in the gut metabolism hypothesis. TMAO and LPS, as markers of gut dysbiosis, activate supplementary signaling cascades, besides inflammation, in the development of atherosclerosis, heart failure, atrial fibrillation, and metabolic disorders. This review summarizes the molecular mechanisms of gut hormones and gut microbiota-derived molecules that affect the properties of vascular cells, cardiomyocytes, β-2 islet cells, and hepatocytes involved in cardiometabolic diseases. Following the described mechanisms, pharmacological agents that show beneficial effects in the pre-clinical experimental studies have not yet been associated with clinical use. Therefore, randomized clinical trials of gut hormones-based therapies in cardiovascular diseases are needed. The design of novel pharmacologic agents that target specific molecular pathways, as those described above, will improve the therapeutic approach to cardiometabolic diseases. 

## Figures and Tables

**Table 1:** An overview of the gut peptides and their main pancreatic effects.

The Gut Molecule	Receptors Activated by the Gut Molecule	The Source of Release	The Pancreatic Effects Produced by the Gut Molecule	References
GLP-1	GLP-1R	Intestinal L cells	Increased insulin biosynthesis and secretion β-cell survival, proliferation, cytoprotection, and differentiation Improved glucose sensibility of β-cell	[9,10,11]
GIP	GIPR	Intestinal K cells	Increased insulin biosynthesis and secretion β-cell survival, proliferation, cytoprotection, and differentiation Improved glucose sensibility of β-cell	[9,11]
Ghrelin/Des-acyl ghrelin	GHS-R1a Ghrelin receptor-like receptors Unacylated ghrelin receptors	A-like gastric oxyntic cells Intestinal cells	β-cell survival Increased insulin secretion	[12]
Peptide YY	Y receptors (Y1, Y4, Y5 receptors)	Intestinal L cells	β-cell proliferation Protection against β-cell apoptosis Insulin sensitivity Insulin secretion via GLP-1 release	[13,14,15]
Neurotensin	NT receptors (NTS1, NTS2, NTS3)	Intestinal neuroendocrine cells	Mediation of the glucose-sensitive insulin secretion Inhibition of the apoptosis of pancreatic β cells	[16]
CCK	CCK1 and CCK2 receptors	Intestinal I cells	Release of pancreatic secretion	[17]

GIP: gastric inhibitory peptide GIPR: GIP receptor; GLP-1: glucagon-like peptide-1; GLP-1R: GLP-1 receptor; GHS-R1a: growth-hormone secretagogue-receptor 1a, NT: neurotensin, CCK: cholecystokinine.

**Table 2:** The main animal studies regarding incretins properties in heart failure.

Gut Molecule	HF Etiology	Study Population	HF Mechanism Influenced	Signaling Pathway Involved	References
GIP	Ang II infusion	Apo E^−/−^ mice Mouse cardiomyocytes	Cardiac hypertrophy, apoptosis, and fibrosis	GIPR/cAMP/phosphorylated Akt axis/downregulation of TGF-β1 and HIF-1α	[160]
Liraglutide, GLP-1 analogue	Ang II infusion and TAC	C57BL6J wild-type mice Rat cardiomyocytes	Cardiac hypertrophy and fibrosis	Inhibition of PI3K/Akt and activation of AMPK	[161]
Liraglutide, GLP-1 analogue	IL-1β infusion	Mouse cardiomyocytes	Cellular oxidative stress, mitochondrial function, and lipid accumulation	AMPK activation	[162]
Liraglutide, GLP-1 analogue	Spontaneous dilated cardiomyopathy	Non-diabetic J2N-k hamsters	Worsened cardiac function and fibrosis	Shortage of glycemic source	[163]
Alogliptin, DPP4 inhibitor	TAC	Male C57BL6 mice Rat cardiomyocytes	Cardiac apoptosis Contractile dysfunction Ventricular remodeling	cAMP/PKA/EPAC1	[166]
GLP-1 infusion	Arterial hypertension	Spontaneously hypertensive, heart failure prone (SHHF) rats	Preserved cardiac function and LV mass Cardiac apoptosis Improved survival Myocardial glucose uptake	Apoptosis proteins	[168]
Miglitol, a-glucosidase inhibitor that increases GLP-1	Arterial hypertension	Dahl salt-sensitive (DS) rats fed a high-salt diet Rat cardiomyocytes	Cardiac dysfunction and remodeling	PKA/mitochondrial-fusion related protein/increased ATP	[169]
GLP-1(28–36), a GLP-1 metabolite	Myocardial ischemia produced by LAD artery ligation	Male C57BL/6J mice Coronary artery endothelial cells Coronary artery smooth muscle cells Mouse cardiomyocytes	Cardiac dysfunction Infarct size Cytoprotection of coronary vascular cells from oxidative injury	Increased ATP/sAC/cAMP/PKA/eNOS phosphorylation MTP-α	[170]
GIPR deletion	Myocardial ischemia produced by permanent LAD coronary artery occlusion	GIP R -/- mice HL-1 cardiac cells	Reduced ventricular ischemic injury Improved survival Increased TAG stores	PKG/ERK/HSL phosphorylation	[171]
Exenatide, a GLP-1 analogue	Myocardial ischemia produced by LAD artery ligation and following reperfusion	Dalland Landrace pigs	Cardiac systolic and diastolic dysfunction Infarct size Nuclear oxidative stress	Akt phosphorylation Bcl2 upregulation, caspase-3 inhibition Increased antioxidant enzymes	[172]
Sitagliptin, a DPP-4 inhibitor	Myocardial ischemia produced by LAD artery ligation	Normoglycemic male Wistar rats Ex vivo-infarcted rat hearts	Arrhythmic risk Sympathetic innervation	GIP/resistin/PI-3K/Akt	[173]
Exendin-4, a GLP-1R agonist	Accumulation of methylglyoxal	Rat cardiomyoblast H9c2 cells	Oxidative stress Apoptosis Mitochondrial dysfunction	cAMP/Epac/PI3K/Akt signaling	[176]
Exendin-4, a GLP-1R agonist	Myocardial ischemia produced by LAD artery ligation	Male Sprague-Dawley rats Cardiac myocytes of adult rats	Infarct size Cardiomyocytes hypertrophy Cardiac dysfunction Chamber dilation and remodeling Cardiac fibrosis Calcium handling	GLP-1 R/eNOS/cGMP/PKG Ca2+/CaMKII Calcium handling proteins	[177]
Exendin-4, a GLP-1R agonist	Myocardial ischemia produced by LAD artery ligation	Adult female C57BL/6J normoglycemic mice Rat ventricular H9c2 cardiomyoblasts and mouse atrial HL-1 cardiomyocytes	Cardiac dysfunction Chamber dilation and remodeling Improved survival Cardiomyocytes hypertrophy and apoptosis Interstitial fibrosis and ECM remodeling Cardiac inflammation	Akt/GSK-3b Smad2/3	[178]
GLP-1(9–36) amide, a metabolically inactive product	Myocardial ischemia produced by LAD artery ligation	Adult female C57BL/6 J mice Rat ventricular H9c2 cardiomyoblasts RAW264.7 murine macrophages	Diastolic dysfunction	ECM turnover Macrophages infiltration	[179]
GLP-1 infusion	HFpEF produced by aortic banding	Male Sprague Dawley rats	Improved survival Diastolic dysfunction Left ventricular stiffness Pulmonary congestion	Metabolic substrate switch toward glucose oxidation	[180]
DPP-4 inhibitor, MK0626	Metabolic cardiomyopathy induced by a high-fat/high-fructose Western diet	C57BL6/J mice	Diastolic dysfunction Cardiac oxidative stress and fibrosis Improved mitochondrial and perivascular ultrastructure		[181]

GIP: gastric inhibitory peptide GIPR: GIP receptor; GLP-1: glucagon-like peptide-1; GLP-1R: GLP-1 receptor; DPP4: dipeptidyl peptidase-4; cAMP: Adenosine 3’,5’-cyclic monophosphate; PKA: protein kinase A; TGF-β1: Transforming Growth Factor-β1; HIF 1α: Hypoxia-inducible factor 1-alpha; AMPK: 5’ adenosine monophosphate-activated protein kinase; PI-3K: Phosphatidylinositol-4,5-bisphosphate 3-kinase; IL-1β: interleukin 1β; Ang II: Angiotensin II, TAC: transverse aortic constriction; EPAC 1: exchange protein directly activated by cAMP 1; MAPK: mitogen-activated protein kinase; LAD: left anterior descendent; sAC: soluble adenyl cyclase; eNOS: endothelial nitric oxide synthase; MTP-α: mitochondrial trifunctional protein-α; Akt-PKB: Akt-protein kinase B; PKC: protein kinase C; BCL-2: B-cell lymphoma 2; PKG: Protein kinase G; ERK: extracellular signal-regulated kinase; CaMKII: calmodulin-dependent kinase II; HSL: hormone-sensitive lipase; GSK-3b: glycogen synthase kinase 3b; HFpEF: heart failure with preserved ejection fraction.

**Table 3:** GLP-1 analogs, GLP-1 receptor agonists (GLP-1RA), and dual GIP/GLP-RA available in 2022 for use in the treatment of T2DM.

Drug Name	Other Important Components	Administration Schedule	Pharmaceutical Company	Efficacy for Glucose Lowering [258]	Efficacy for Weight Loss [258]	References
For Subcutaneous Injection						
Exenatide * Byetta Bydureon BCise	Encapsulation of Exenatide in Poly-(d,l-Lactide-Co-Glycolide)	Twice daily Once weekly	AstraZeneca AstraZeneca	High High	Intermediate Intermediate	[259,260]
Lixisenatide Lyxumia(EU) Adlyxin(USA)	Six lysine tail	Once daily	Sanofi	High	Intermediate	[261]
Liraglutide Victoza	Free fatty acid promoting binding to albumin	Once daily	Novo Nordisk	High	High	[262]
Dulaglutide Trulicity	Immunoglobulin Fc fragment	Once weekly	Eli Lilly & Co.	High to very high	High	[263]
Semaglutide Ozempic	Free fatty acid promoting binding to albumin	Once weekly	Novo Nordisk	Very high	Very high	[262]
Tirzepatide Mounjaro	C20 fatty diacid promoting binding to albumin	Once weekly	Eli Lilly & Co.	Very high	Very high	[264]
Fixed combination						
Liraglutide/insulin degludec (iDegLira) Xultophy	Basal insulin	Once daily	Novo Nordisk	Very high	NA **	[265]
Lixisenatide/insulin glargine (iGlarLixi) Soliqua (USA) Suliqua (EU)	Basal insulin	Once daily	Sanofi	Very high	NA **	[266]
For oral administration						
Semaglutide Rybelsus	Free fatty acid promoting binding to albumin SNAC	Once daily	Novo Nordisk	High to very high	High to very high	[267]

GLP-1—Glucagon-like peptide-1; GIP—glucose-dependent insulinotropic polypeptide; * Interestingly, was observed that exendin-4 is an antagonist of the GIP receptors [267]. ** NA—not applicable for weight loss, due to combination with insulin; SNAC—sodium N-[8-(2-hydroxybenzoyl) amino caprylate.

## Data Availability

Not applicable.
